# Structure-Aware OCTA Segmentation for OCTA-Derived Quantitative Analysis of Cerebral Microvascular Perfusion in Ischemic Stroke

**DOI:** 10.3390/tomography12080110

**Published:** 2026-07-29

**Authors:** Jingmin Luan, Tian Jiang, Xiaohao Sun, Jian Liu, Yao Yu, Xiaojiao Dong, Yang Wang, Yajie Zhang, Zhenhe Ma

**Affiliations:** 1 School of Computer and Communication Engineering, Northeastern University at Qinhuangdao, No. 143 Taishan Road, Qinhuangdao 066004, China; luanjingmin@neuq.edu.cn (J.L.); 202312127@stu.neuq.edu.cn (T.J.); 202312007@stu.neuq.edu.cn (X.S.); 2 School of Intelligent Sensing and Optoelectronic Engineering, Northeastern University at Qinhuangdao, No. 143 Taishan Road, Qinhuangdao 066004, China; 1000972@neuq.edu.cn (J.L.); yuyao@neuq.edu.cn (Y.Y.); 3 Department of Neurology, The First Hospital of Qinhuangdao, Qinhuangdao 066000, China; gracedongxj@163.com (X.D.); 18603392616@wo.cn (Y.W.); zhangyajiedoctor@163.com (Y.Z.)

**Keywords:** medical image segmentation, optical coherence tomography angiography, cerebral microvascular quantification, vascular perfusion density, ischemic stroke

## Abstract

**Background:** Ischemia-related optical coherence tomography angiography (OCTA) signal degradation complicates cerebral vessel segmentation and quantitative analysis. We developed a structure-aware segmentation framework and applied it to exploratory longitudinal vascular quantification. **Methods**: Six adult male C57BL/6 mice were imaged at baseline and 0.5–10 h after photothrombotic induction. After geometric transformation, 345 image instances were separated by animal into training, validation, and test subsets of 240, 30, and 75 images from four, one, and one mouse; the 75 test instances originated from 25 source images. A separate sixfold subject-level cross-validation generated out-of-sample masks for longitudinal analysis. **Results**: The proposed method achieved a Dice coefficient of 0.887, a centerline Dice (clDice) of 0.953, a branch-point F1 score of 0.739, and an average surface distance (ASD) of 0.564 pixels, with significant improvements in Dice, clDice, and branch-point F1. Vascular perfusion density (VPD) and vessel length (VL) decreased significantly over time, vessel average diameter (VAD) remained stable, and average curvature (AC) and branch-point density (BPD) showed significant overall temporal effects. **Conclusions**: The framework improved technical segmentation performance and supported exploratory analysis of OCTA-derived vascular measurements in experimental ischemic stroke.

## Simple Summary

Stroke can weaken vascular signals in optical coherence tomography angiography (OCTA), making vessel segmentation and derived measurements less reliable. We developed a structure-aware cerebral OCTA segmentation method and evaluated it in a photothrombotic mouse model. The method improved overlap, boundary, centerline, and branch-point agreement and was tested under controlled image degradation. In a separate longitudinal analysis, out-of-sample masks showed reduced OCTA-detectable perfusion coverage and apparent vessel length after ischemia, while average vessel diameter changed little. This exploratory preclinical study characterizes OCTA-detectable vascular changes after experimental ischemic stroke.

## 1. Introduction

Ischemic stroke remains a major cause of mortality and long-term neurological disability worldwide [1,2]. Although large-vessel recanalization has improved acute stroke treatment, restoration of macrovascular patency does not necessarily ensure recovery of the cerebral microcirculation [3,4,5]. Persistent microvascular dysfunction may contribute to impaired reperfusion and progression of ischemic injury [6,7]. Penumbral evolution, microvascular constriction, and impaired capillary reflow are central components of acute ischemic injury [8,9,10], while high-resolution optical methods support repeated visualization of microvascular networks [11,12,13,14]. Photothrombotic mouse models combined with OCTA therefore provide a practical platform for longitudinal cerebral microvascular imaging [14,15]. Quantitative indices derived from OCTA can characterize detectable vascular patterns, but their reliability depends on accurate vessel segmentation.

Ischemia-related signal attenuation, blurred vessel boundaries, and local signal discontinuities make cerebral OCTA segmentation challenging [16,17,18,19]. Region-overlap metrics such as the Dice coefficient quantify pixel-wise agreement but do not fully characterize boundary displacement, centerline continuity, or bifurcation preservation. These structural errors may affect downstream skeleton-derived measurements, particularly vessel length, branch-point density, and average curvature. Therefore, segmentation of ischemic cerebral OCTA images requires accurate regional delineation together with reliable representation of fine vessels and vascular network structure.

In this study, “structural stability” means agreement with the reference annotation in boundary localization [95th-percentile Hausdorff distance (HD95) and average surface distance (ASD)], centerline continuity [centerline Dice (clDice) and centerline precision/recall], branch-point preservation (branch-point F1), and connected-component organization. It does not mean scan–rescan or longitudinal repeatability.

Traditional vascular-segmentation methods mainly rely on intensity thresholding and multi-scale vesselness filtering [20,21]. Encoder–decoder convolutional networks and transformer-based architectures subsequently improved feature representation and multi-scale contextual modeling [22,23,24]. General-purpose and medical foundation segmentation models have further provided transferable representations for medical image segmentation [25,26,27,28,29], whereas structure- and boundary-aware networks have introduced task-specific constraints for curvilinear anatomical structures [30,31]. However, the integration of these complementary strategies for ischemic cerebral OCTA, together with their evaluation in relation to downstream vascular quantification, remains limited. To address this need, we developed a structure-aware vessel-segmentation framework based on a lightweight Swin-LiteMedSAM backbone. The framework integrates structure-guided boundary distillation (SBD), a geometry-aware boundary attention mechanism (GBAM), a multi-scale deep supervision strategy (MDSS), and a multi-scale collaborative attention module (MCAM).

This study comprised two analytically distinct components. First, a technical segmentation experiment evaluated the proposed method against representative segmentation approaches using subject-level data separation and complementary overlap-, boundary-, centerline-, and bifurcation-sensitive metrics. Second, a separate exploratory longitudinal application used out-of-sample masks generated by sixfold subject-level cross-validation to calculate vascular perfusion density (VPD), vessel length (VL), vessel average diameter (VAD), branch-point density (BPD), and average curvature (AC) in a photothrombotic mouse stroke model.

The main contributions of this study are as follows:

- A structure-aware cerebral OCTA segmentation framework that integrates boundary distillation, geometry-aware attention, multi-scale supervision, and cross-scale feature refinement within Swin-LiteMedSAM.
- An evaluation protocol combining overlap, boundary, centerline, bifurcation, and connected-component metrics with source-image-level statistics, controlled degradation tests, ablation, and parameter-sensitivity analysis.
- An exploratory longitudinal pipeline using out-of-sample masks to calculate five OCTA-derived vascular measurements after photothrombotic ischemia.

## 2. Related Works

### 2.1. Fine-Structure Vascular Segmentation Methods and Quantitative Limitations

Thin branching vessels are sensitive to noise, low contrast, and local signal loss. Thresholding and vesselness filtering are efficient but vulnerable to these changes [20,21], whereas U-Net and transformer models learn stronger representations [22,23,24]. Dice and intersection over union (IoU) may still miss boundary displacement, centerline breaks, and false connections that alter skeleton-derived length, branch density, and curvature [32,33,34]. Ischemic OCTA therefore requires both region- and structure-sensitive evaluation.

### 2.2. Structure-Aware, Boundary-Aware, and Multi-Scale Segmentation Strategies

Boundary losses improve edge localization, skeleton or topology losses encourage continuity, clDice measures centerline–region agreement, multi-scale supervision helps retain fine vessels, and attention modules refine feature selection [30,31,35,36,37,38]. In the proposed network, SBD supplies annotation-derived guidance during training, GBAM adds geometric boundary cues, MDSS supervises intermediate scales, and MCAM refines cross-scale features. These components are structure-sensitive but do not guarantee topology preservation or biomarker reproducibility.

Table 1 compares these strategies and their relevance to ischemic OCTA quantification.

### 2.3. General-Purpose and Foundation Segmentation Models

Foundation models such as SAM and medical lightweight derivatives provide transferable representations and efficient backbones [25,26,27,28,29]. However, their general segmentation objectives are not specific to weak cerebral OCTA boundaries or skeleton-sensitive vascular structures. We therefore retained Swin-LiteMedSAM as the backbone and added OCTA-specific structural constraints rather than using a larger generic architecture.

### 2.4. OCTA-Based Microvascular Visualization and Quantification Studies

OCT/OCTA enables repeated, high-resolution visualization of microvascular networks and supports the measurement of perfusion density, vessel length, vessel diameter, branch density, and tortuosity [13,14,16,39,40,41]. Recent OCTA segmentation studies have explored weak supervision, connectivity-aware modules, and multi-scale feature learning [42,43,44], whereas cerebral OCTA studies have mainly focused on characterizing perfusion-related changes after experimental stroke [15,45,46]. Because reported segmentation performance is strongly influenced by anatomy, the retinal or cerebral layer analyzed, dataset composition, image quality, and annotation protocol, cross-study Dice values should be interpreted as contextual references rather than direct performance rankings.

Previous studies have generally examined either segmentation accuracy or biological changes derived from OCTA, with relatively limited attention to the technical reliability of the intermediate segmentation and skeletonization steps. The present study therefore separates the two analytical components: segmentation experiments are used to evaluate the technical performance of the proposed method, while the longitudinal analysis applies out-of-sample masks to provide an exploratory description of OCTA-derived vascular changes after experimental stroke.

## 3. Materials and Methods

**Figure 1:**
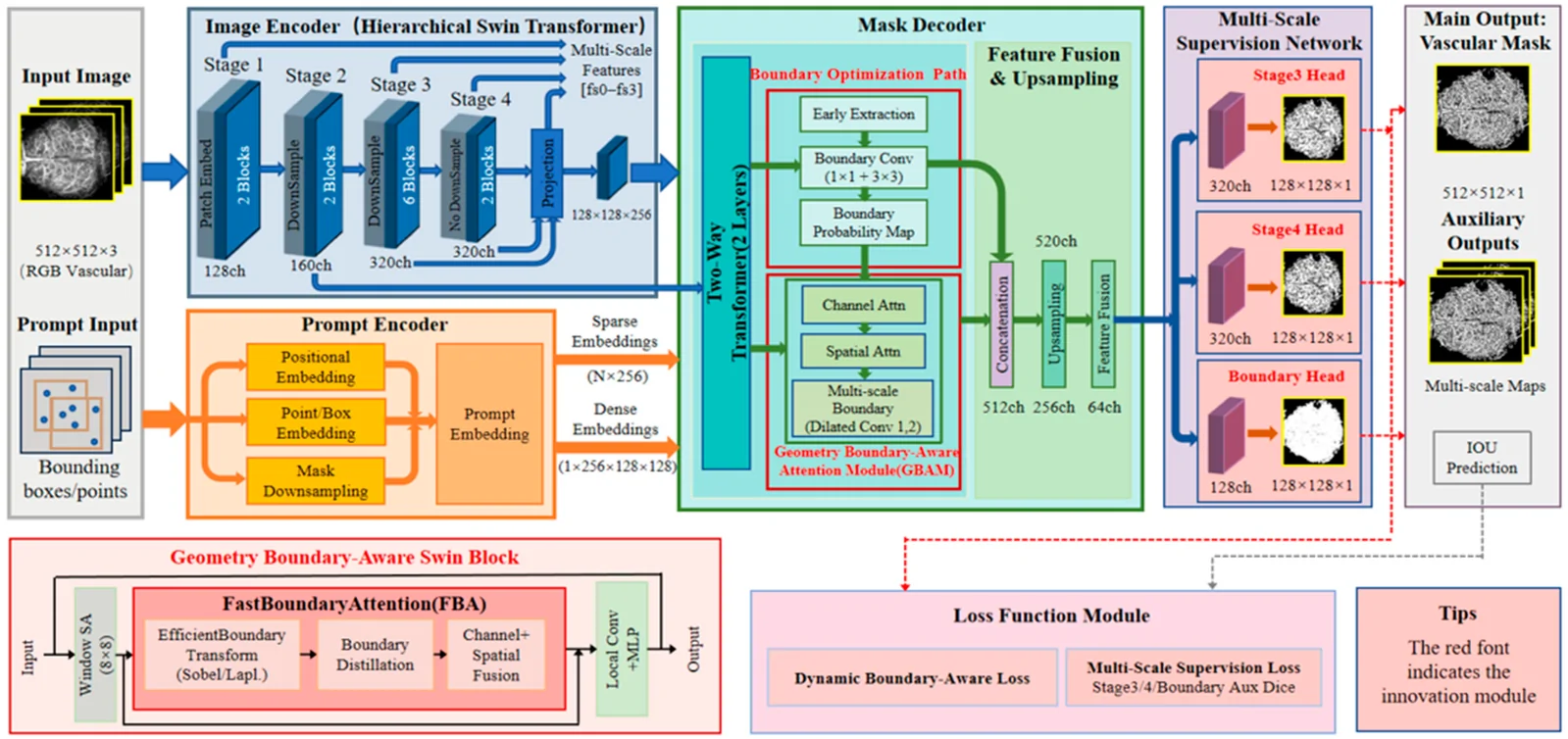
Overview of the proposed segmentation and quantification framework. Abbreviations: RGB, red, green, and blue; Conv, convolution; Attn, attention; MLP, multilayer perceptron. Blue solid arrows indicate image-feature propagation and feature transfer between the image encoder, mask decoder, and multi-scale supervision network; orange solid arrows indicate prompt-embedding propagation; green solid arrows indicate boundary-optimization and feature-fusion flow within the mask decoder; and black solid arrows indicate feature processing within the geometry boundary-aware Swin block. Red dashed arrows indicate auxiliary-output and supervision-loss connections, whereas gray dashed arrows indicate the IoU-prediction connection. The light-blue, orange, light-green, purple, gray, red-outlined pink, light-pink, and peach boxes denote the image encoder, prompt encoder, mask decoder, multi-scale supervision network, output block, geometry boundary-aware Swin block, loss-function module, and explanatory note, respectively. Red text identifies the proposed innovation modules.

To address common degradation issues in ischemic cerebral OCTA images, including reduced vascular contrast, blurred boundaries, and microvascular discontinuities, we construct a fully automated segmentation and quantitative analysis framework. The framework adopts the lightweight prompt-based segmentation model Swin-LiteMedSAM as the backbone network. Multidimensional structural constraints and semantic enhancement mechanisms are introduced to improve the modeling capability for complex degraded vascular morphologies while preserving computational efficiency. Figure 1 shows an overview of this framework.

### 3.1. Data Acquisition and Preprocessing

All procedures were approved by the Institutional Animal Care Committee of Northeastern University and followed the Guide for the Care and Use of Laboratory Animals. The final cohort comprised six adult male C57BL/6 mice (20–30 g); their exact age was unavailable in the original records. Inclusion required successful induction, baseline imaging, complete longitudinal acquisition, and adequate OCTA signal. Prespecified exclusions were failed induction, uncorrectable motion, poor signal, incomplete imaging, or procedure-related death; none of these occurred. The available cohort was used without an a priori sample-size calculation.

Photothrombosis was induced under sodium pentobarbital anesthesia (sodium pentobarbital, Sigma-Aldrich, St. Louis, MO, USA) by tail-vein Rose Bengal disodium salt injection (Rose Bengal, Sigma-Aldrich, St. Louis, MO, USA, formulated to 20 mg/mL in physiological saline) and irradiation with a 532 nm solid-state laser (Model MGL-III-5 mW, CNI Laser, Changchun, Jilin, China) for 30 min. OCTA was acquired at baseline and at 0.5, 1, 1.5, 2, 3, 4, 6, 8, and 10 h. A customized swept-source optical coherence tomography system with a central wavelength of 1310 nm, self-developed at Northeastern University at Qinhuangdao, provided 7.5 μm axial and 16 μm lateral resolution over approximately 8 × 7.5 mm; A-scan-wise motion correction and combined differential/decorrelation processing generated OCTA images [39,40,41,47].

Filtered and normalized optical attenuation coefficient (OAC) maps were calculated from structural OCT [48]. Ipsilateral pixels below the subject-specific 25th percentile of the contralateral OAC distribution defined the low-attenuation region of interest (ROI). This rank-based imaging criterion reduced inter-animal signal differences but was not histologically validated and was not interpreted as an ischemic core.

### 3.2. Annotation and Dataset Construction

Reference masks were prepared in Fiji v1.54f (bundled ImageJ 1.54f, National Institutes of Health, Bethesda, MD, USA). One trained researcher annotated each image; a second reviewed vessel continuity, boundaries, fine vessels, artifacts, and bifurcations; and unresolved cases were adjudicated by a senior OCT/OCTA researcher. The consensus mask served as ground truth.

Three experts, blinded to animal identity, time point, experimental condition, and annotator identity, rated annotation accuracy, boundary adherence, and topological plausibility on a five-point scale (5, excellent; 4, good; 3, acceptable; 2, poor; 1, unacceptable). Specific scoring criteria are detailed in Table S1 of the Supplementary Materials. Each expert reviewed all 115 original, non-augmented OCTA image–mask pairs; geometrically transformed derivatives were not rescored. The audit was descriptive quality control rather than an objective repeat-annotation study. The mean overall expert score was 4.60 ± 0.35 under the stated criteria.

For each animal and time point, the OAC-defined ROI comprised ipsilateral pixels below the 25th percentile of the corresponding contralateral OAC distribution:

(1) $${\mathrm{ROI}}_{\mathrm{low}\text{-}\mathrm{OAC}}=\{x\in H_{\mathrm{ipsi}}|\mathrm{OAC}\left(x\right)<Q_{0.25}\left({\mathrm{OAC}}_{H_{\mathrm{contra}}}\right)\}$$

$Q_{0.25}$ was prespecified as a rank-based within-subject threshold. It was not treated as a pathological cutoff, and other percentiles were not tested.

Two trained readers, blinded to the time point and vascular measurements, independently corrected obvious artifacts in the automatically generated ROIs; disagreements were resolved by consensus or senior adjudication. Interobserver consistency was assessed on 20 original images spanning all six mice and the full imaging period (Table 2). These were technical samples, not biological replicates.

Predefined flips and mirror transformations produced 345 instances from 115 original OCTA images. Four mice were assigned to training (240 images), one to validation (30), and one to testing (75). The 75 test instances represented three geometrically related versions of 25 source images and were treated as clustered observations.

Method comparison used a fixed subject-level split, whereas longitudinal quantification used out-of-sample masks from sixfold subject-level cross-validation; the two statistical analyses were kept separate.

For longitudinal masks, sixfold subject-level cross-validation held out one mouse for testing and one for validation in each fold; every mouse served once as the test animal, producing out-of-sample masks for all six mice.

### 3.3. Statistical Analysis

Segmentation and longitudinal analyses were separate. For segmentation inference, the 75 test-instance metrics were averaged by source-image identifier to yield 25 paired observations. Friedman tests with Kendall’s W compared seven methods for Dice, clDice, branch-point F1, and connected-component relative error. Two-sided Wilcoxon signed-rank tests compared the proposed method with each baseline, with Holm correction; cluster-bootstrap 95% confidence intervals (10,000 source-image resamples) and rank-biserial correlations were reported.

For longitudinal analysis, image measurements were averaged within mouse and time point, making the mouse the independent unit (*n* = 6). VPD, VL, VAD, and BPD were normalized to baseline; AC remained on its original scale. Overall effects used one-way repeated-measures ANOVA with Greenhouse–Geisser correction and generalized η^2^. Prespecified baseline comparisons used two-sided paired *t*-tests with Holm–Bonferroni correction within each biomarker; mean differences, unadjusted 95% confidence intervals, and Cohen’s dz were reported. The available cohort and wide intervals support exploratory interpretation.

### 3.4. Implementation Details and Evaluation Metrics

Subject-level partitioning preceded augmentation, and all methods used the same split and training conditions. A fixed full-field 512 × 512 bounding-box prompt was generated automatically without ground-truth information or manual interaction.

Conventional metrics were Dice, IoU, recall, HD95, and ASD; pixel accuracy was descriptive because background dominance can inflate it. Structure-sensitive metrics used identical binarization, post-processing, Zhang–Suen skeletonization, and 8-connectivity settings.

Let *P* and *G* denote the predicted and reference masks and *S*(·) denote skeletonization. Centerline precision, centerline recall, and clDice were defined as follows:

Centerline precision was the fraction of the predicted skeleton within the reference mask:

(2) $$P_{\mathrm{cl}}=\frac{\left|S\left(P\right)\cap G\right|}{\left|S\left(P\right)\right|+\varepsilon }$$

Centerline recall was the fraction of the reference skeleton covered by the prediction:

(3) $$R_{\mathrm{cl}}=\frac{\left|S\left(G\right)\cap P\right|}{\left|S\left(G\right)\right|+\varepsilon }$$

clDice was their harmonic mean:

(4) $$\mathrm{clDice}=\frac{2P_{\mathrm{cl}}R_{\mathrm{cl}}}{P_{\mathrm{cl}}+R_{\mathrm{cl}}+\varepsilon }$$

Branch points were detected on one-pixel skeletons using 8-neighborhood connectivity; adjacent candidates were merged and junctions matched one-to-one within 3 pixels. The harmonic mean of branch-point precision and recall was reported as branch-point F1.

Global fragmentation was measured by connected-component relative error:

(5) $$E_{\mathrm{CC}}=\frac{\mathrm{abs}\left(N_{P}-N_{G}\right)}{\max\left(N_{G},1\right)}$$

where $N_{P}$ and $N_{G}$ are the numbers of 8-connected components. Metrics from all 75 instances were aggregated to 25 source-image values for inference.

Comparators were Otsu, U-Net, Swin-Unet, TransUNet, UNeXt, and HiFormer.

All model training, inference, image processing, skeleton extraction and quantitative vascular analysis were performed using Python 3.12, PyTorch 2.1.2, CUDA 12.1, OpenCV-Python 4.8.1, NumPy 1.26, and Matplotlib 3.8. Statistical hypothesis testing was conducted using SciPy 1.11; SPSS 28.0 (IBM, Armonk, NY, USA) was used for supplementary group statistics. The Fiji v1.54f MorphLibJ v1.4.7 plugin was utilized for morphological operations and skeletonization.

Efficiency was measured on an NVIDIA RTX 4060 with 512 × 512 inputs, a batch size of 1, and 32-bit floating-point (FP32) precision. After 50 warm-up iterations, 200 forward passes were averaged, excluding loading, transfer, and disk input/output. The model contained 48.99 million parameters and required 44.0 ms/image (22.7 frames per second [FPS]); unmeasured multiply–accumulate operations (MACs), floating-point operations (FLOPs), memory, training time, and baseline latency are not reported as measured quantities.

Controlled degradation used the fixed 75-image test subset without retraining or changes to prompts, thresholds, or post-processing.

Contrast scaling was defined as

(6) $$I_{c}=\mu +c\left(I-\mu \right)$$

where c = 0.80 or 0.60. Gaussian noise was defined as

(7) $$I_{n}=\mathrm{clip}\left(I+\varepsilon ,0,1\right),\;\;\varepsilon ∼N\left(0,\sigma^{2}\right)$$

where σ = 0.02 or 0.05; Gaussian blur used σ = 1 or 2 pixels, and motion kernels had lengths of 5 or 9 pixels. Robustness was measured using the following metrics: Dice, clDice, ASD, and branch-point F1. The relative decreases were calculated as

(8) $$\frac{M_{\mathrm{clean}}-M_{\mathrm{degraded}}}{M_{\mathrm{clean}}}\times 100\%$$

For ASD, the relative increase was calculated as

(9) $$\frac{M_{\mathrm{degraded}}-M_{\mathrm{clean}}}{M_{\mathrm{clean}}}\times 100\%$$

### 3.5. Network Architecture and Methodology

The architecture targets four common ischemic OCTA failure modes: weak boundaries, geometry-dependent boundary ambiguity, scale-related loss of fine vessels, and low-signal semantic confusion. Accordingly, SBD provides training-time structural guidance, GBAM modulates geometry-sensitive boundary features, MDSS constrains intermediate scales, and MCAM refines cross-scale semantics. These modules are task-specific constraints rather than a formal topology guarantee.

The encoder produces multi-scale features {*x*_0_,…,*x*_4_}; structural and geometric modulation is applied during feature extraction, auxiliary heads supervise intermediate representations, and MCAM refines decoded features before the final mask is generated.

#### 3.5.1. Structure-Guided Boundary Distillation Module (SBD)

SBD transfers structural information from the reference mask to intermediate features during training. Let *F* ∈ R^(B×C×H×W) be an encoder feature map, and let *M* be the corresponding mask.

*M* is downsampled to the resolution of *F* and partitions vessel and non-vessel feature vectors.

Specifically, the reference mask was resized to the spatial resolution of the feature map, denoted by $\tilde{M}$. Vessel and non-vessel features were then defined as *F_v_* = *F*⊙$\tilde{M}$ and *F_nv_* = *F*⊙(1 − $\tilde{M}$), respectively, where ⊙ denotes element-wise multiplication with spatial broadcasting across channels.

Cross-attention between the two partitions produces a boundary-difference representation:

(10) $$Q\;\;=\;\;W_{q}F_{v}\;,\;K\;\;={\;\;W}_{k}F_{\mathrm{nv}}\;,\;V\;\;=\;{\;W}_{v}F_{\mathrm{nv}}$$

(11) $$\mathrm{Attention}(Q,K,V)=\mathrm{softmax}(\frac{QK^{T}}{\sqrt{d_{k}}})V\;$$

where $W_{q}$, ${\;W}_{k}$, and ${\;W}_{v}$ are the learned projections and $d_{k}$ is the key dimension.

The enhanced teacher feature is fused residually:

(12) $$F_{T}=F+\alpha \cdot BDC(F,M)$$

Here, α = σ (a) is a learnable feature-fusion coefficient that controls the contribution of the mask-guided boundary representation to the teacher feature. It was initialized at 0.50 by setting a_0_ = 0 and was constrained to (0, 1) through the sigmoid function.

A student feature *F*_S_, generated without mask-guided decomposition, was trained using

(13) $$L_{\mathrm{distill}}=\frac{1}{\mathrm{BCHW}}\left|\right|F_{S}-\mathrm{sg}(F_{T})|{|}_{F}^{2}$$

Here, $F_{S}$ denotes the corresponding feature generated by the mask-free student branch, and sg(∙) denotes the stop-gradient operation. The normalization by BCHW makes the magnitude of the distillation loss less dependent on feature-map size. Gradients from *L*_distill_ were propagated only through the student branch, whereas the mask-guided teacher feature served as a detached structural target.

The mask-guided teacher branch is active only during training. At inference, the OCTA image alone enters the student encoder–decoder; SBD therefore adds no test-time input or computation.

#### 3.5.2. Geometry-Aware Boundary Attention Mechanism (GBAM)

GBAM combines learned features with lightweight Sobel, Laplacian, and morphological-gradient cues. These operators act as boundary-oriented priors rather than standalone segmentation rules and are combined as follows:

(14) $$G_{\mathrm{combined}}\;\;=\;\;(G_{\mathrm{sobel}}\;\;+\;\;G_{\mathrm{laplacian}}\;\;+\;\;G_{morph})\;/\;3\;$$

Directional non-maximum suppression yields a refined response, followed by the confidence map:

(15) $$P_{\mathrm{boundary}}\;\;=\;\;\sigma \;(\beta \cdot (G_{\mathrm{suppressed}}-\tau \;))$$

The response was normalized to [0, 1]. The fixed parameters β = 10 and τ = 0.50 controlled the transition steepness and activation level.

The confidence map modulates channel-attended features:

(16) $$F_{\mathrm{enhanced}\;}\;=\;\;F_{\mathrm{ca}}\;+{\;F}_{\mathrm{ca}}⊙P_{\mathrm{boundary}}\cdot \lambda$$

The bounded coefficient λ = σ (l) was initialized at 0.50 and learned within (0, 1).

GBAM was inserted within Swin Transformer blocks to propagate boundary cues across feature levels.

#### 3.5.3. Multi-Scale Deep Supervision Strategy (MDSS)

MDSS attaches auxiliary heads to Stage 3, Stage 4, and an intermediate decoder boundary feature to constrain semantic, spatial, and boundary representations.

The auxiliary loss was

(17) $$L_{\mathrm{aux}}\;=\;\sum_{i=1}^{3}\gamma_{i}(t)\cdot L_{\mathrm{Dice}}(P_{i},M_{\mathrm{gt}}^{i})$$

where $P_{i}$ is the *i*-th auxiliary prediction and $\gamma_{i}(t)$ is its scheduled weight.

The coefficient schedule was

(18) $$\gamma_{i}\left(t\right)=\left(1-\frac{t}{T}\right)\gamma_{i}\left(0\right)+\frac{t}{T}\gamma_{i}(T)$$

where *t*/*T* denotes the normalized training progress.

The weights decayed linearly from (0.30, 0.20, 0.30) to (0.10, 0.10, 0.10) for the three heads.

Thus, auxiliary supervision was strongest early and progressively reduced during main-branch refinement.

#### 3.5.4. Multi-Scale Collaborative Attention Module (MCAM)

MCAM aligns decoded multi-scale features and applies collaborative channel–spatial attention to strengthen vessel-related semantics in low-signal or locally blurred regions.

### 3.6. Loss Function and Training Strategy


(19) $$L_{boundary}=1-\frac{2\left\langle B(P),B(M)\right\rangle +\varepsilon }{{\left\|B(P)\right\|}_{1}+{\left\|B(M)\right\|}_{1}+\varepsilon }.$$

The boundary loss was calculated as a Dice-type discrepancy between the boundary maps extracted from the predicted probability map and the reference mask, where B(·) denotes the boundary-extraction operator and ε is a numerical-stability constant.

The primary segmentation loss was

(20) $$L_{\mathrm{main}}\;\;={\;w}_{d}\cdot L_{\mathrm{Dice}}\;\;+\;w_{b}\cdot L_{\mathrm{BCE}}\;+\;w_{\mathrm{bd}}\;(t)\;\cdot L_{\mathrm{boundary}}\;$$

$L_{\mathrm{Dice}}$ addressed foreground imbalance,$\cdot L_{\mathrm{BCE}}$ stabilized pixel classification, and $L_{\mathrm{boundary}}$ measured the Dice agreement between the predicted and reference boundary maps.

The fixed weights were ${\;w}_{d}$ = 0.6 and $w_{b}$ = 0.2.

The boundary coefficient followed

(21) $$w_{\mathrm{bd}}\;(t)\;=\;0.20\;\;+\;0.40\;\frac{t}{T}$$

The boundary-loss coefficient increased linearly from 0.20 to 0.60 during training, limiting excessive boundary dominance during early optimization while progressively strengthening boundary refinement.

The total objective was

(22) $$L={\;L}_{\mathrm{main}}+L_{\mathrm{aux}}+w_{\mathrm{distill}}\cdot L_{\mathrm{distill}}$$

When SBD was enabled, w_distill_ was fixed at 0.10 across all experiments and random seeds; it was neither learned nor dynamically scheduled. In configurations without SBD, $L_{\mathrm{distill}}$ was omitted. The coefficient *α* in Equation (12) controls feature-level fusion within the teacher branch, whereas w_distill_ controls the contribution of the distillation term to the total optimization objective.

Training was performed end to end. ${\;L}_{\mathrm{main}}$ was calculated from the final student prediction, *L_aux_* was calculated from the three intermediate predictions, and $L_{\mathrm{distill}}$ aligned the mask-free student feature with the detached mask-guided teacher feature. The teacher and auxiliary branches were used only during training. At inference, the reference mask was unavailable, and only the mask-free student segmentation branch was retained; therefore, SBD introduced no additional inference input or teacher-branch computation.

Sensitivity was assessed one factor at a time using three seeds: *α*/*λ* initializations of 0.25, 0.50, or 0.75; β ∈ {5, 10, 20} and τ ∈ {0.30, 0.50, 0.70}; fixed or linearly scheduled boundary weights; and low, high, or linearly decayed MDSS weights. Dice, clDice, ASD, branch-point F1, final *α*/*λ*, and failed runs were recorded.

### 3.7. Collaborative Integration of Structure- and Semantics-Oriented Modules

SBD, GBAM, and MDSS impose structure-oriented constraints, whereas MCAM refines semantic fusion through shared features and the joint loss. “Collaborative” denotes this architectural integration, not a proven internal optimization mechanism; configuration-dependent effects were tested by ablation.

## 4. Results

### 4.1. Comparison with State-of-the-Art Methods

#### 4.1.1. Qualitative Results

**Figure 2:**
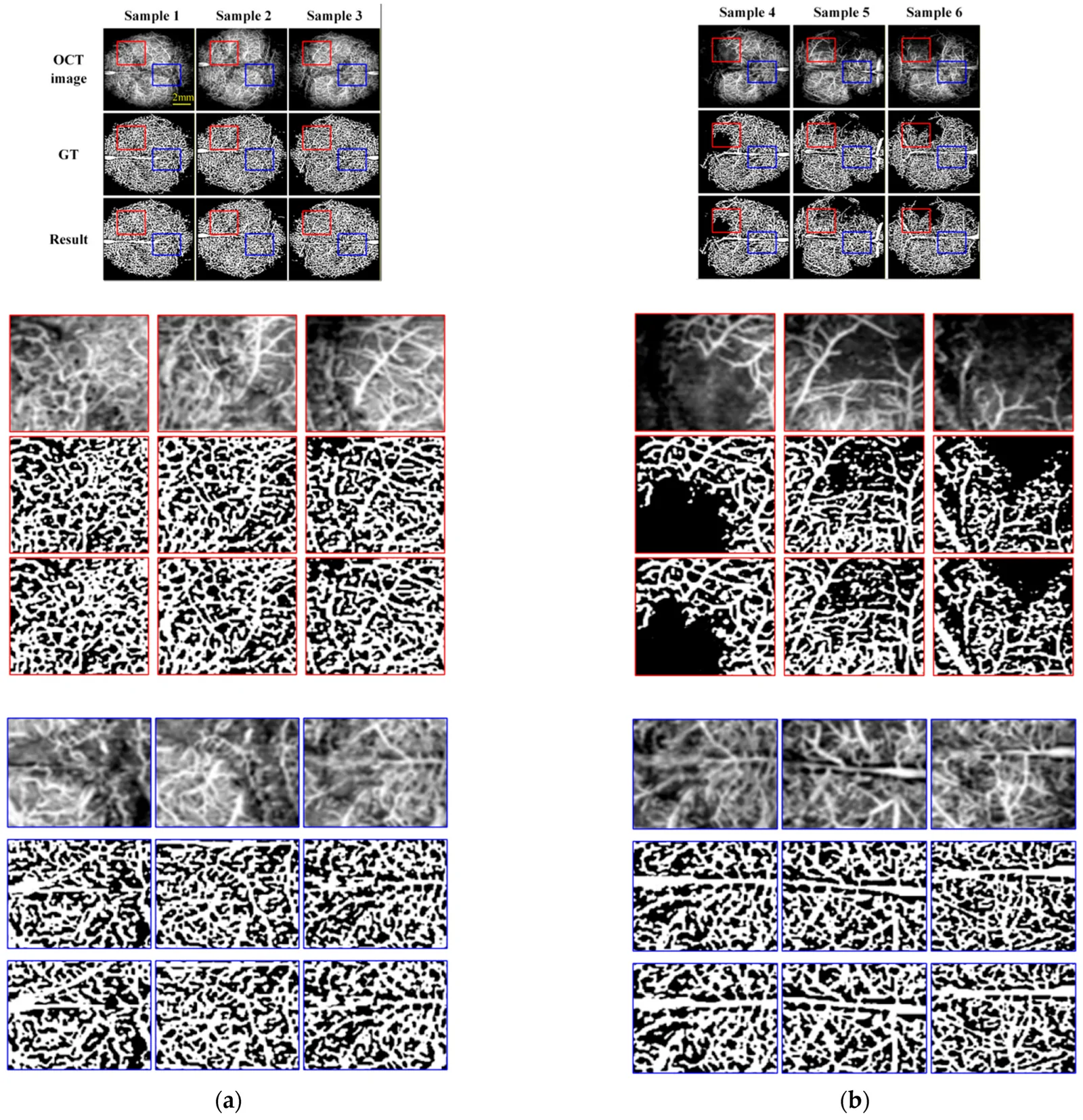
Representative full-field and enlarged vessel-segmentation results in the non-ischemic and ischemic hemispheres: (**a**) samples 1–3 show representative non-ischemic regions; (**b**) samples 4–6 show representative ischemic regions. Columns represent individual samples. The first three rows show the original optical coherence tomography (OCT) images, ground-truth masks (GT), and predictions of the proposed method, respectively. The middle three rows show the enlarged OCT image, GT, and prediction corresponding to the red ROI, while the final three rows show the corresponding enlargement of the blue ROI. The red and blue squares indicate the regions enlarged in the middle and bottom panels, respectively.

Figure 2 presents illustrative test cases selected to show visually distinct non-ischemic and ischemic OCT appearances. The displayed cases were chosen from the fixed held-out test subset based on the visibility of the corresponding imaging phenotype: non-ischemic cases showed relatively preserved vascular contrast and network appearance, whereas ischemic cases showed evident signal attenuation, reduced vascular visibility, or local discontinuity.

**Figure 3:**
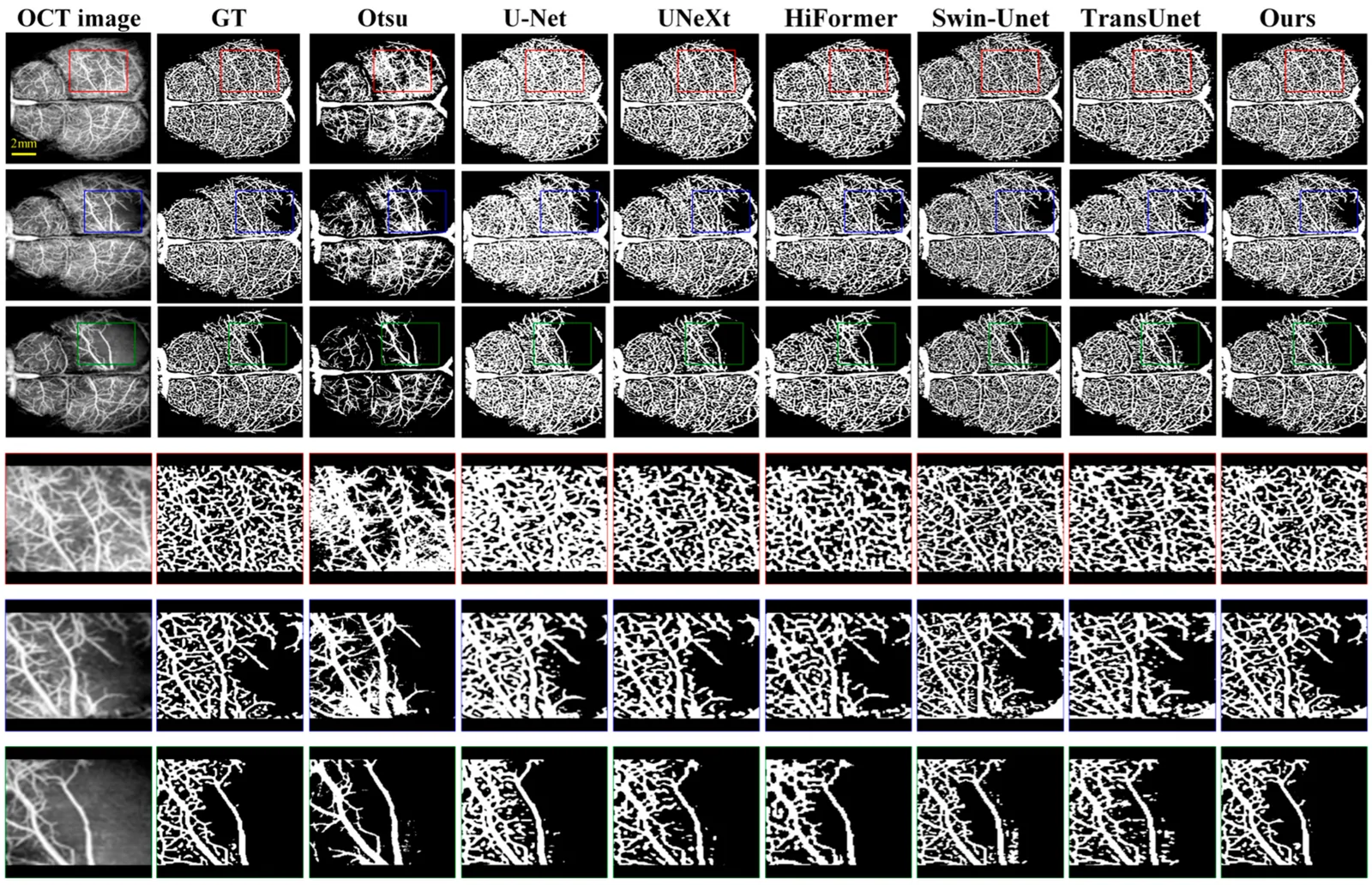
Qualitative comparison of representative segmentation methods with matched ROI enlargements. Columns from left to right show the original OCT image, ground-truth mask (GT), Otsu, U-Net, UNeXt, HiFormer, Swin-Unet, TransUNet, and the proposed method. Row 1 shows the full-field results, with the red ROI marking a dense fine-vessel region. Row 2 shows the same full-field results, with the blue ROI highlighting a major vessel trunk and its adjacent branches. Row 3 shows the same full-field results, with the green ROI emphasizing vessel-boundary delineation and nearby small branches. Rows 4, 5, and 6 present the corresponding enlarged red, blue, and green ROIs, respectively. Abbreviations: Otsu, Otsu thresholding; U-Net, U-shaped convolutional neural network; UNeXt, MLP-based rapid medical image segmentation network; HiFormer, hierarchical multi-scale transformer; Swin-Unet, Swin Transformer-based U-Net; TransUNet, transformer-based U-Net.

Figure 3 presents a qualitative comparison of the proposed method and representative segmentation approaches using the same full-field OCT image. Three matched ROIs were selected to show fine vessels, major branches, and ambiguous boundaries; identical coordinates and magnification were used for the OCT image, ground truth, and every method. The examples were illustrative, while conclusions were based on the full test subset.

Qualitative comparison showed that Otsu missed many fine vessels, while several learning-based methods exhibited local discontinuities, false connections, or boundary thickening. Although Swin-Unet improved in these aspects, it still fell short in boundary handling. The proposed method more closely matched the ground truth, with better preservation of fine vessels, vascular continuity, and boundary details.

Across the three selected ROIs, the proposed method showed closer qualitative agreement with the ground truth in fine-vessel continuity and local branch organization, although some local segmentation discrepancies remained.

#### 4.1.2. Quantitative Comparison on the Test Set

All methods used the same four-mouse training, one-mouse validation, and one-mouse test split; the test set contained 75 transformed instances from 25 source images.

**Figure 4:**
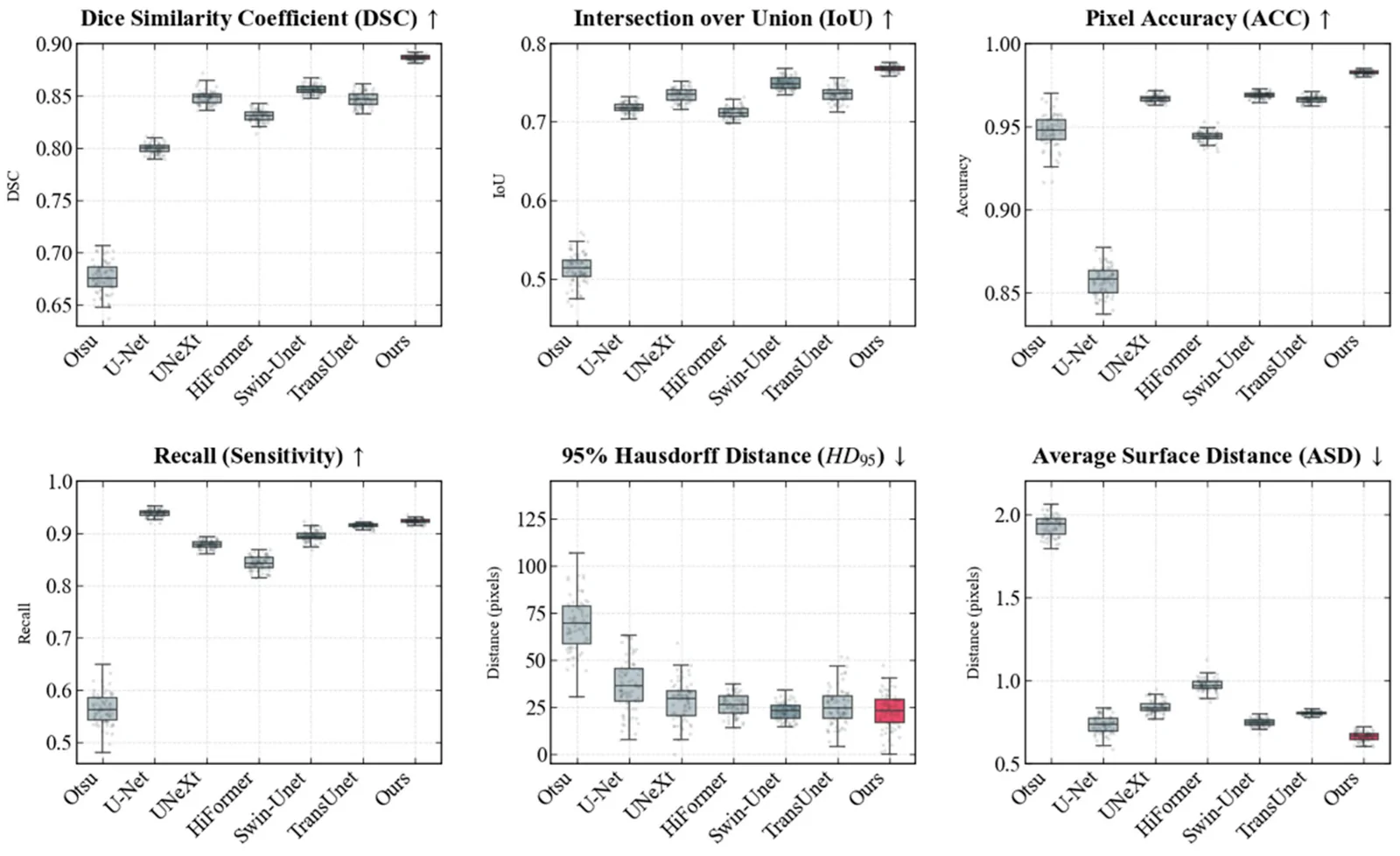
Box plot distributions of segmentation performance metrics over the fixed test subset containing 75 transformed OCTA images from one held-out test mouse. Upward arrows indicate that higher values represent better performance, whereas downward arrows indicate that lower values represent better performance.

The proposed method achieved the highest Dice, IoU, and accuracy and the lowest ASD (Table 3; Figure 4) but not the highest recall or the lowest HD_95_. U-Net retained more vessel pixels, and Swin-Unet had slightly fewer extreme boundary outliers. The main gain was a better balance of overlap and average boundary agreement.

Because overlap and boundary metrics do not directly quantify vessel-centerline continuity, bifurcation preservation, or network fragmentation, topology- and centerline-specific results are reported separately in Table 4.

The proposed method achieved the highest clDice, centerline precision/recall, and branch-point F1. Its connected-component relative error was lower than those of the five comparators but slightly higher than that of Swin-Unet, indicating improved centerline and branch agreement rather than uniform superiority across all connectivity measures.

The proposed method achieved a connected-component relative error of 0.201 ± 0.020, which was lower than that of Otsu, U-Net, UNeXt, HiFormer, and TransUnet but slightly higher than that of Swin-Unet (0.196 ± 0.023). Thus, Swin-Unet showed the closest agreement with the reference in terms of connected-component count, whereas the proposed method showed the best centerline–region consistency and branch-point agreement. Taken together, these results support improved topology-related structural consistency, particularly in vascular centerline coverage and bifurcation preservation, rather than uniform superiority in every connectivity-related measure.

Friedman tests and Holm-corrected Wilcoxon comparisons were performed on 25 paired source-image-level observations; the strongest comparator for each metric is summarized in Table 5.

Method effects were significant for Dice, clDice, branch-point F1, and connected-component relative error (all *p* < 0.001; Kendall’s W = 0.639–0.828). Against the strongest competitor, improvements were 0.032 for Dice (95% CI 0.023–0.040; adjusted *p* = 3.60 × 10^−5^; r = 0.871), 0.012 for clDice (0.006–0.018; adjusted *p* = 0.00140; r = 0.711), and 0.040 for branch-point F1 (0.025–0.054; adjusted *p* = 2.40 × 10^−4^; r = 0.791). Connected-component error did not differ from Swin-Unet (benefit −0.005, 95% CI −0.013 to 0.002; adjusted *p* = 0.491). All source images came from one held-out mouse, so these are internal technical results.

#### 4.1.3. Sixfold Subject-Level Cross-Validation for Out-of-Sample Mask Generation

A separate sixfold subject-level procedure generated out-of-sample masks for longitudinal analysis: each mouse served once as the test animal, one other mouse was used for validation, and four were used for training. Fold Dice ranged from 0.876 to 0.888. The fold-specific models were applied to all images of their held-out mouse, yielding masks for all six animals.

#### 4.1.4. Robustness to Prompt Perturbation and Controlled Image Degradation

Prompt robustness was first evaluated using the bounding-box perturbations reported in Table 6. Dice remained between 0.880 and 0.887 under the tested prompt settings, whereas ASD ranged from 0.564 to 0.615. These results indicate that moderate bounding-box perturbations had a limited influence on region-overlap performance, although small variations in boundary localization remained detectable.

**Figure 5:**
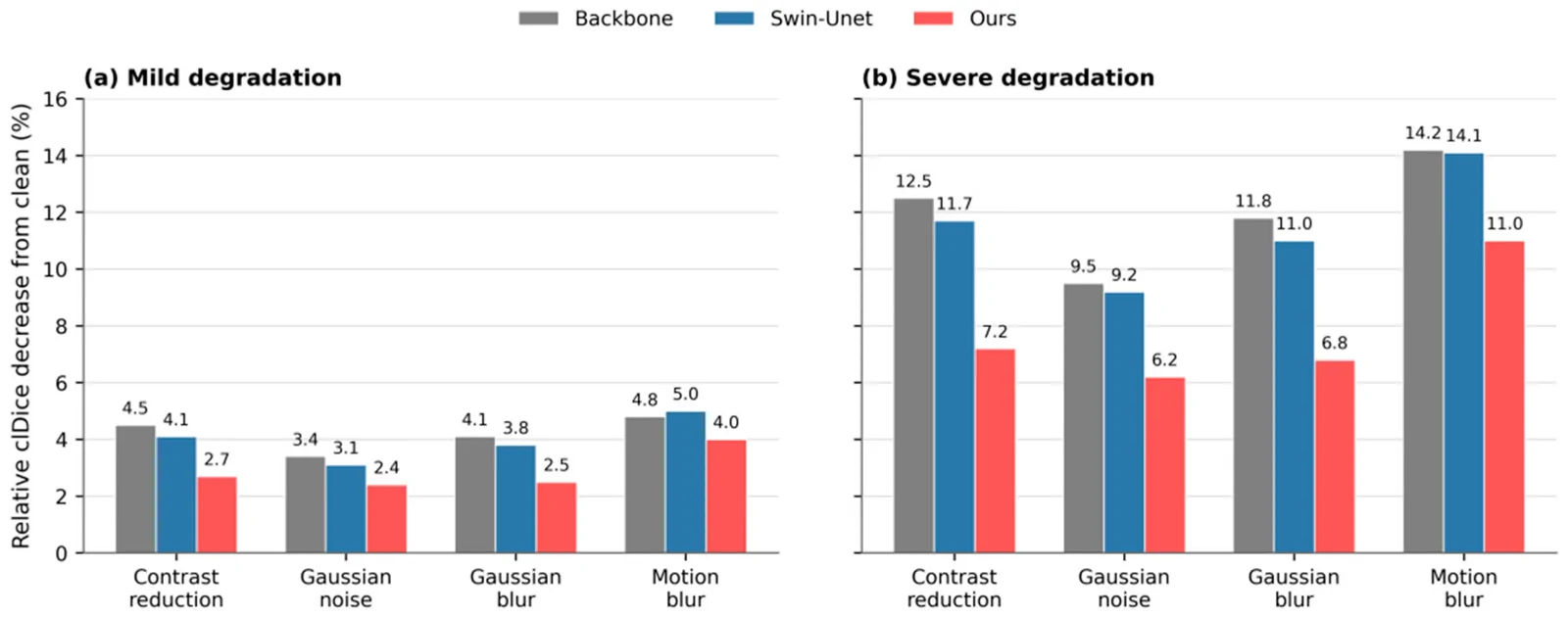
Relative clDice decreases under controlled OCTA image degradation. Relative changes from the corresponding clean condition are shown for the Swin-LiteMedSAM backbone, Swin-Unet, and the proposed model under (**a**) mild and (**b**) severe contrast reduction, Gaussian noise, Gaussian blur, and linear motion blur. Lower values indicate greater robustness to controlled image degradation. The complete Dice, clDice, ASD, and branch-point F1 results are provided in Table S2 of the Supplementary Materials.

To complement the prompt-level evaluation, robustness to controlled OCTA image degradation was further examined using contrast reduction, Gaussian noise, Gaussian blur, and linear motion blur. All three models showed progressive performance deterioration as degradation severity increased. Averaged across the four mild degradation types, the relative clDice decreases were 4.2% for the Swin-LiteMedSAM backbone, 4.0% for Swin-Unet, and 2.9% for the proposed model. Under severe degradation, the corresponding decreases were 12.0%, 11.5%, and 7.8%, respectively. The degradation-specific relative clDice changes are shown in Figure 5.

The proposed model had the smallest clDice decrease across all eight conditions. Severe motion blur was most disruptive, lowering Dice from 0.8870 to 0.8107 and clDice from 0.9530 to 0.8482; the clDice decrease was 11.0%, versus 14.2% and 14.1% for the backbone and Swin-Unet.

### 4.2. Ablation Study and Model Analysis

All ablations used the same backbone, module locations, subject-level split, and training configuration. Table 7 varied only in the inclusion of SBD, GBAM, MDSS, and MCAM. The sensitivity of the coefficients is analyzed in Section 4.2.3, and the loss plans are presented in Table S3 of the Supplementary Materials.

#### 4.2.1. Single-Module Analysis

Among the individual modules, SBD produced the largest improvement, increasing Dice from 0.8478 to 0.8800 while reducing HD95 from 33.89 to 25.64 pixels and ASD from 0.712 to 0.588 pixels. This broad improvement across overlap and boundary metrics is consistent with the role of SBD as the only module that directly introduces annotation-guided structural information into intermediate feature learning during training.

GBAM produced a more modest improvement in Dice and ASD, suggesting that geometry-based modulation mainly refined the existing feature representation rather than providing an additional structural training target. MDSS produced only a small Dice increase but improved recall and HD95, indicating a greater contribution to vascular-region sensitivity and localized boundary control than to overall overlap.

MCAM alone increased recall but worsened Dice and ASD. Thus, cross-scale semantic refinement alone appeared to recover additional vessel pixels at the cost of regional and boundary precision. Overall, SBD provided the strongest individual contribution, whereas GBAM, MDSS, and MCAM primarily affected different aspects of boundary refinement, sensitivity, and semantic fusion.

#### 4.2.2. Multi-Module Analysis

The multi-module effects were not strictly additive. Although SBD alone achieved a Dice of 0.8800, SBD+GBAM decreased Dice to 0.8595 and increased both HD95 and ASD. Other partial configurations containing SBD also did not consistently retain the improvement obtained with SBD alone. These results indicate that the effect of structure-guided features depended on their subsequent modulation and supervision within the joint configuration.

In contrast, GBAM+MDSS achieved better Dice, HD95, and ASD than either corresponding single-module configuration. This pattern suggests a complementary effect underlying the tested setting, because geometry-related feature modulation and intermediate multi-scale supervision acted at different stages of the segmentation pathway.

MCAM-containing partial configurations generally increased recall without corresponding improvements in Dice or boundary-distance metrics. For example, GBAM+MCAM achieved the highest recall among the partial configurations but also showed relatively poor HD95 and ASD. These combinations therefore altered the balance between vessel-pixel sensitivity and boundary precision rather than producing uniform improvements.

The complete model achieved the highest Dice and the lowest HD95 and ASD, although it did not achieve the highest recall. Its principal advantage was therefore a more favorable overall balance among overlap, sensitivity, and boundary consistency. Taken together, the results show that module contributions were configuration-dependent rather than strictly additive. These interpretations describe the observed metric patterns in relation to the designed roles of the modules and do not constitute direct evidence of an internal optimization mechanism.

The present ablation study evaluated the inclusion and selected combinations of the four principal modules. Parameter-sensitivity analyses additionally examined selected coefficient initializations and loss-weight schedules. The backbone, module locations, subject-level data split, and training configuration were held fixed across the module-ablation experiments. Accordingly, the results characterize module contributions and configuration-dependent interactions within the defined architecture.

#### 4.2.3. Parameter Sensitivity and Training Stability

**Figure 6:**
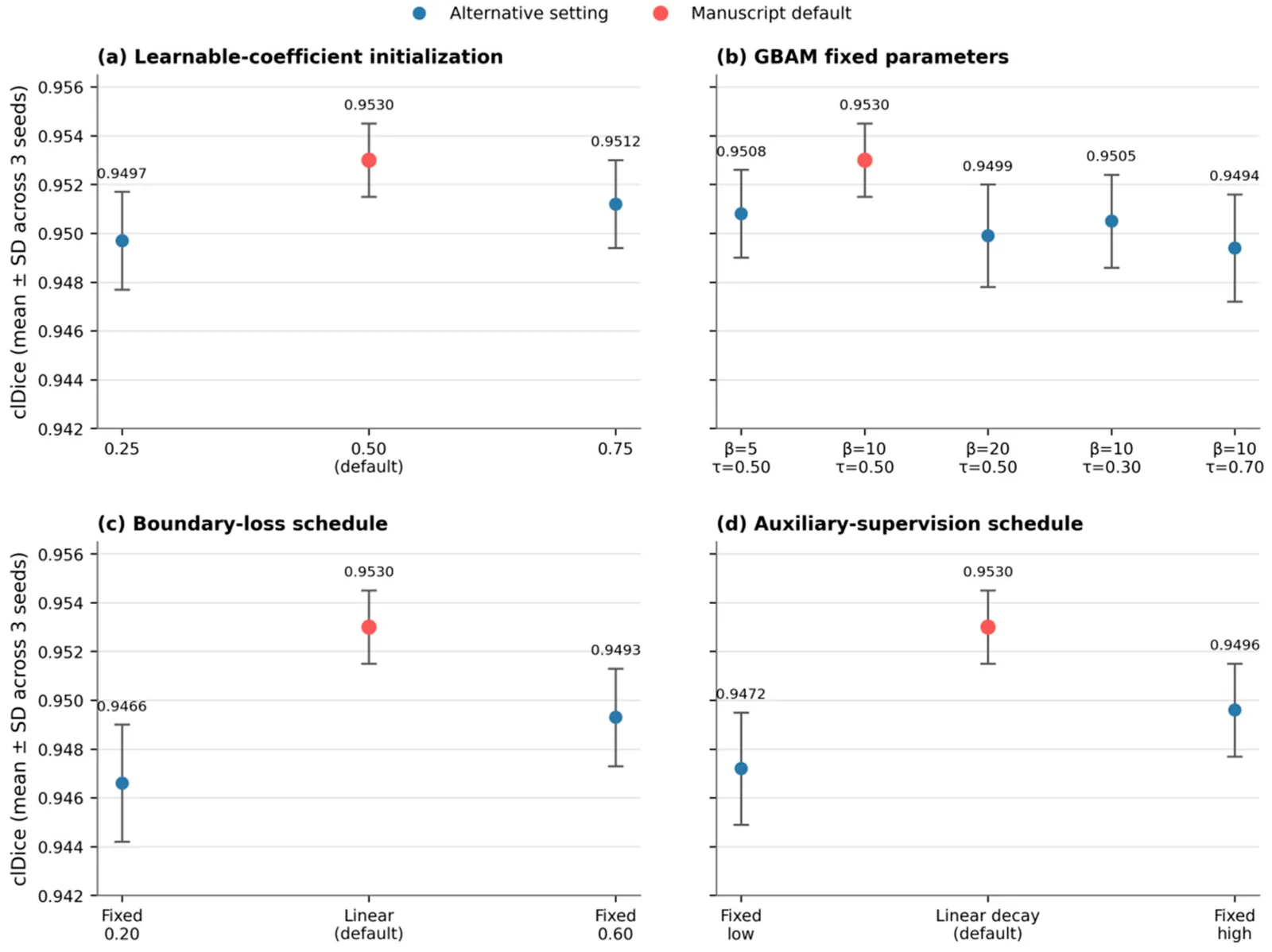
Sensitivity of clDice to learnable-coefficient initialization, fixed GBAM parameters, and dynamic loss-weight schedules. Points indicate the mean clDice across three independent training seeds, and error bars indicate the corresponding standard deviation. (**a**) Joint initialization of the bounded learnable coefficients *α* and *λ*; (**b**) fixed GBAM slope and threshold parameters *β* and *τ*; (**c**) boundary-loss weighting strategy; and (**d**) auxiliary-supervision weighting strategy. The manuscript-default configuration is highlighted in red. The complete results for Dice, clDice, ASD, branch-point F1, the final learned coefficients, and the failed runs are provided in Table S3 of the Supplementary Materials.

Figure 6 summarizes the three-seed tests of coefficient initialization, fixed GBAM parameters, boundary-loss scheduling, and auxiliary-supervision scheduling.

No runs failed. Across *α*_0_/*λ*_0_ values of 0.25–0.75, Dice varied from 0.8854 to 0.8870 and clDice from 0.9497 to 0.9530; final *α* and *λ* remained bounded, indicating low sensitivity to moderate initialization changes.

GBAM sensitivity was also modest. Although *β* = 5 gave a marginally higher Dice, *β* = 10 and *τ* = 0.50 provided the best combined clDice, ASD, and branch-point F1 and were retained as the balanced setting.

The linear boundary schedule (0.20 → 0.60) outperformed fixed low or high weights in clDice, ASD, and branch-point F1, indicating that progressively stronger boundary emphasis was preferable to a constant weight.

Likewise, linearly decayed MDSS weights produced a better balance of clDice, ASD, and branch-point F1 than fixed low or high weights, despite only small differences in Dice.

Overall, all tested settings were numerically stable; the larger changes in structure-sensitive metrics compared with Dice show that these choices mainly affected boundary and centerline behavior rather than region overlap.

#### 4.2.4. Computational Efficiency

The proposed model contains approximately 48.99 million parameters. Under the specified NVIDIA RTX 4060 testing environment, the average inference time was 44.0 ms per OCTA image, corresponding to 22.7 FPS. The structure-guided boundary distillation module and the auxiliary-supervision branches are used only during training and therefore do not introduce additional computation during standard inference.

### 4.3. Longitudinal OCTA-Derived Vascular Quantification After Ischemic Stroke

Longitudinal vascular quantification was performed as a separate exploratory application using out-of-sample masks generated by sixfold subject-level cross-validation. All parameters were calculated within the same prespecified OAC-defined low-attenuation ROI workflow to maintain consistent regional selection across time points.

#### 4.3.1. Vascular Network Quantification Pipeline

To enable automated extraction of multidimensional structural parameters from segmentation results, a standardized quantification pipeline was established as follows:

- Mask Post-processing and Skeleton Extraction

Morphological closing and hole filling were applied, components <50 pixels were removed, and Zhang–Suen thinning generated one-pixel centerlines.

Skeletonization was tested on 100 synthetic phantoms under clean, mild, and severe perturbations against Lee thinning and medial-axis extraction. Algorithm dependence was also examined on 20 original reference/prediction pairs spanning all six mice and imaging phases; these were technical samples, not biological replicates.

- Morphological Parameter Calculation

To quantitatively characterize the cerebral microvascular network from the segmentation results, several vascular parameters were calculated based on the binary vessel masks and their corresponding skeleton representations. The definitions of these metrics are described below.

**Vascular Perfusion Density (VPD).** VPD was defined as the proportion of vascular pixels within the ROI:

(23) $$\mathrm{VPD}\;=\frac{N_{\mathrm{vessel}}}{N_{\mathrm{ROI}}}\times 100\%$$

**Vessel Length (VL).** Total vessel length was calculated by counting skeleton pixels and converting them to physical scale using system calibration parameters:

(24) $$\mathrm{VL}\;\;=\;\;N_{\mathrm{skeleton}}\;\times {\;d}_{\mathrm{pixel}}$$

where $N_{\mathrm{skeleton}}$ denotes the number of skeleton pixels and $d_{\mathrm{pixel}}$ represents the physical pixel spacing.

**Vessel Average Diameter (VAD).** A Euclidean distance transform was applied to the binary mask. Local radii were extracted at skeleton locations, and corresponding diameters were computed as follows:

(25) $$D_{i}\;\;=\;\;2R_{i}$$

The regional average vessel diameter was obtained by averaging diameters across all skeleton points.

**Average Curvature (AC).** Skeleton curves were reconstructed as ordered point sequences. Local curvature was calculated for each skeleton point using the discrete geometric relationship among adjacent triplets of points. The average curvature was defined as the mean curvature value across all skeleton points within the ROI, providing a global descriptor of vascular tortuosity.

**Branch-Point Density (BPD).** Branch points were detected based on 8-neighborhood topology. A pixel was defined as a branch point when the number of foreground neighbors exceeded two. Branch-point density was calculated as follows:

(26) $$\mathrm{BPD}\;\;=\;\;\frac{N_{\mathrm{branch}}}{A_{\mathrm{ROI}}}$$

where $N_{\mathrm{branch}}$ is the number of branch points and $A_{\mathrm{ROI}}$ denotes the ROI area.

- Regional Statistics and Temporal Normalization

All parameters were calculated separately within concentric analysis zones. To reduce the influence of inter-individual baseline variability, VPD, VL, VAD, and BPD were normalized relative to baseline (0 h before ischemia induction) according to

(27) $$P_{\mathrm{norm}}(t)\;=\;\frac{P(t)}{P\left(0\right)}\;\times \;100\%$$

where P(t) denotes the parameter value at time t and P(0) represents the baseline value before ischemia induction. AC was analyzed using its original values without normalization, as its magnitude already reflects the intrinsic geometric characteristics of the vascular skeleton.

- Sensitivity analysis of skeleton-derived parameters

To assess the robustness of BPD and AC to segmentation and skeleton artifacts, 30 original, non-augmented OCTA images were selected by prespecified stratified sampling from the six mice across the 0–10 h period. For each image, the ground-truth mask and the corresponding sixfold out-of-sample prediction were analyzed as a paired set. The 30 images were treated as technical image-level samples rather than independent biological replicates.

The analysis was limited to BPD and AC because they depend directly on junction detection and local skeleton geometry. Spatially adjacent branch-point candidates were merged before BPD calculation to avoid duplicate counting. This experiment characterized selected perturbation sensitivities and was not intended as complete uncertainty propagation for all vascular biomarkers.

- Baseline repeatability

Baseline repeatability was assessed using two independent pre-ischemia OCTA acquisitions from each mouse, with repositioning and redefinition of the imaging window before the second scan. Identical imaging, ROI, segmentation, and post-processing procedures were applied. The first acquisition served as the normalization reference, whereas the second was used only for repeatability assessment.

Repeatability was evaluated using ICC(A, 1), within-subject coefficients of variation, Bland–Altman bias and 95% limits of agreement, and repeatability coefficients.

#### 4.3.2. Temporal Morphological Changes in the Cerebral Vascular Network

**Figure 7:**
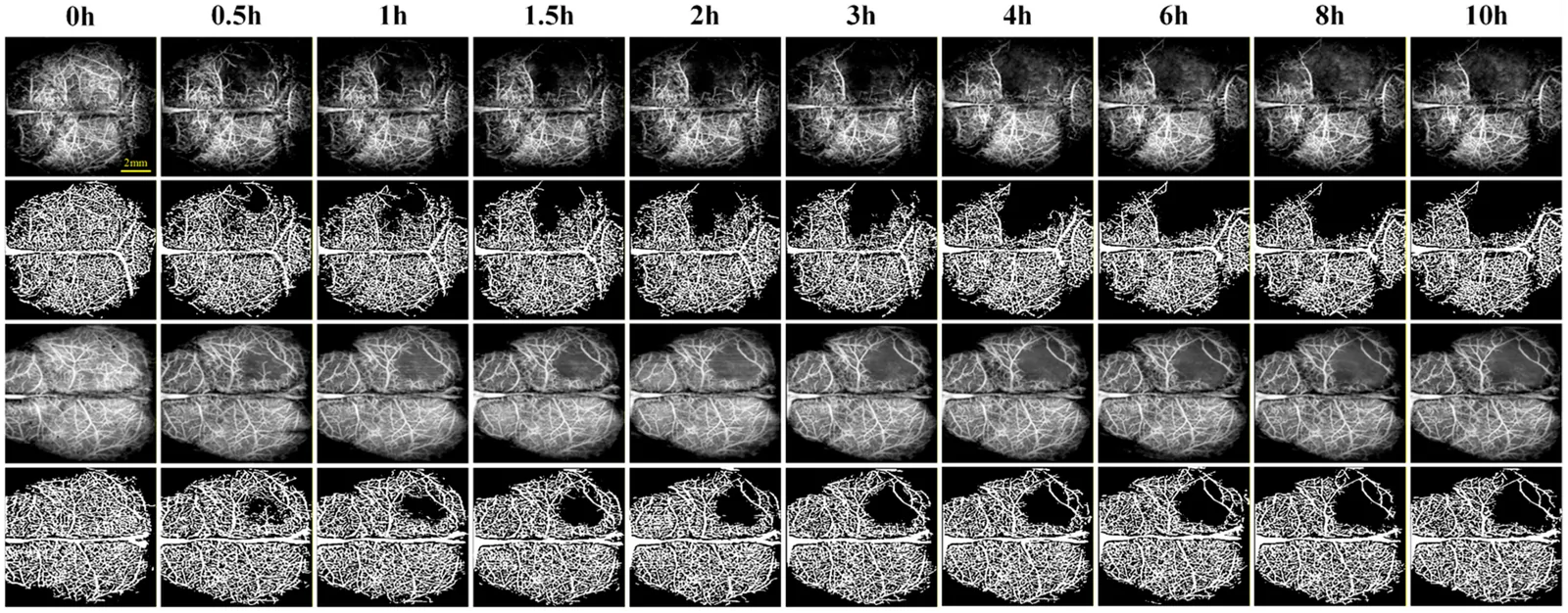
Temporal morphological changes in the cerebral vascular network from baseline to 10 h after ischemia induction.

During longitudinal imaging, the vascular network exhibited stage-dependent morphological changes after ischemia induction (Figure 7). In the early phase, major vascular trunks and most branching structures remained relatively intact. Over time, signal intensity in the ischemic region decreased, accompanied by reduced continuity of fine vessels and localized sparsification. Around 10 h post-induction, the morphology tended to stabilize, although overall perfusion coverage remained lower than at baseline.

#### 4.3.3. Changes in Cerebral Perfusion and Structural Parameters

**Figure 8:**
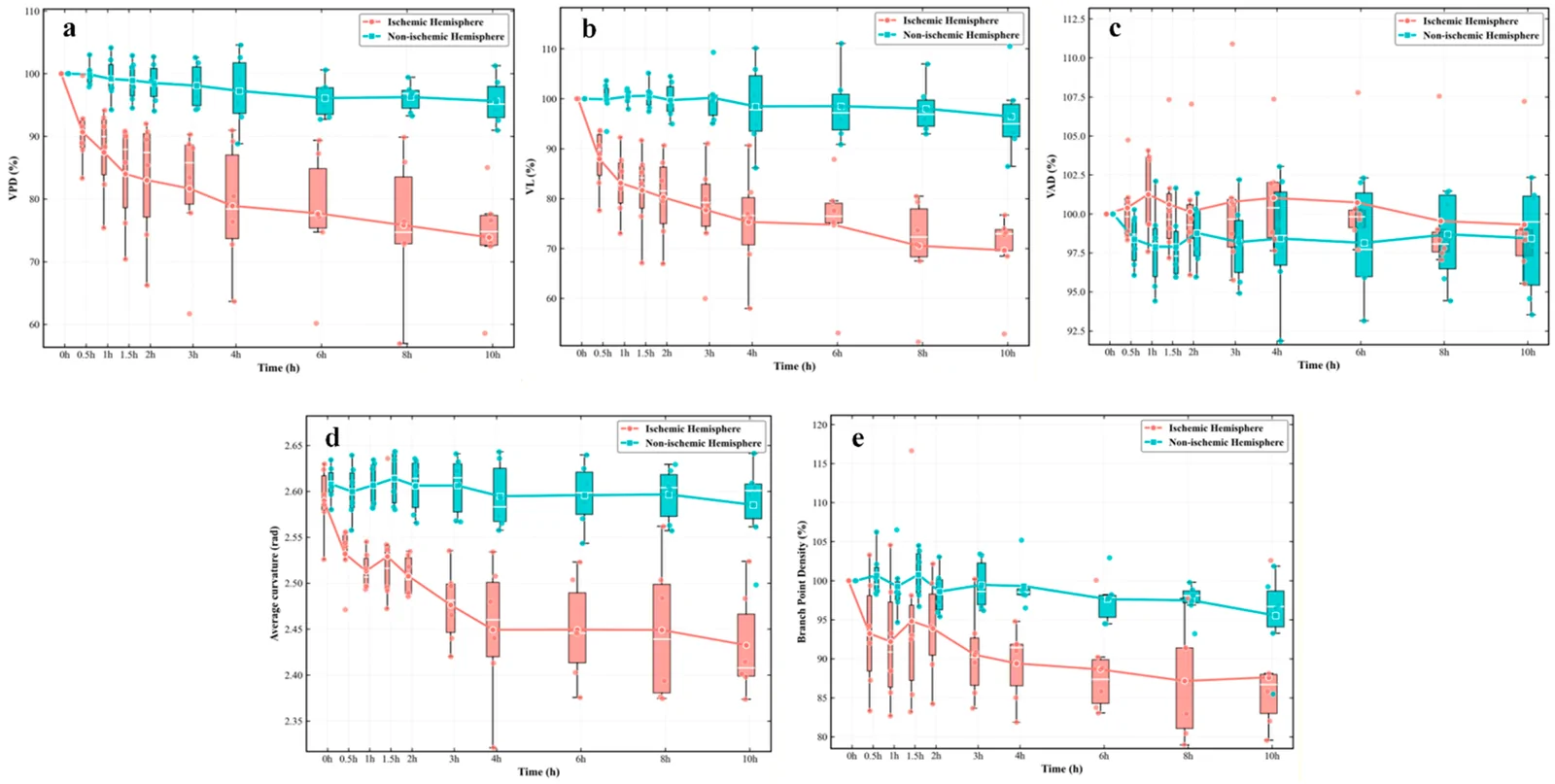
Longitudinal changes in vascular quantitative metrics in the ischemic and non-ischemic hemispheres: (**a**) VPD, (**b**) VL, (**c**) VAD, (**d**) AC, (**e**) BPD. Boxes indicate the interquartile range, central horizontal lines indicate the median, whiskers extend to values within 1.5 times the interquartile range, and individual points represent the six mice. Colored lines connect the arithmetic mean at each time point.

To systematically evaluate post-stroke microvascular structural changes, five vascular parameters were longitudinally compared between ischemic and non-ischemic hemispheres (Figure 8).

In the ischemic hemisphere, VPD and VL decreased in the early phase and remained at lower levels thereafter, indicating reduced OCTA-detectable perfusion coverage and apparent vascular length. BPD and AC also showed gradual declines, suggesting possible changes in the apparent topology of the segmented OCTA vascular network.

In contrast, VAD exhibited only minor fluctuations, indicating that major vessel calibers remained relatively stable. In the non-ischemic hemisphere, all parameters showed only small fluctuations without consistent trends.

Taken together, the decrease in VPD and VL likely reflects ischemia-related impairment of OCTA-detectable microvascular perfusion. This change may be associated with reduced flow visibility, a weakened perfusion signal, and possibly structural vascular alterations. However, because OCTA vessel visibility is affected by flow velocity, signal intensity, decorrelation thresholding, and motion artifacts, reduced VPD should not be interpreted as direct evidence of anatomical vessel loss or irreversible structural simplification without further validation.

Repeated-measures analysis (Table 8) showed significant effects for VPD (F(3.069, 15.344) = 16.197, *p* < 0.001, and generalized η^2^ = 0.449) and VL (F(3.113, 15.564) = 18.401, *p* < 0.001, and generalized η^2^ = 0.522); all baseline comparisons remained significant after Holm correction. VAD was not significant (*p* = 0.601). AC and BPD showed significant overall effects (*p* = 0.001 and 0.013), but only AC at 6 h and BPD at 4 h remained significant after correction. The independent experimental unit was the mouse (*n* = 6).

Complete baseline-to-post-ischemia paired comparisons, including mean differences, unadjusted 95% confidence intervals, Holm-adjusted *p* values, and paired effect sizes, are provided in Table S4 of the Supplementary Materials.

**Figure 9:**
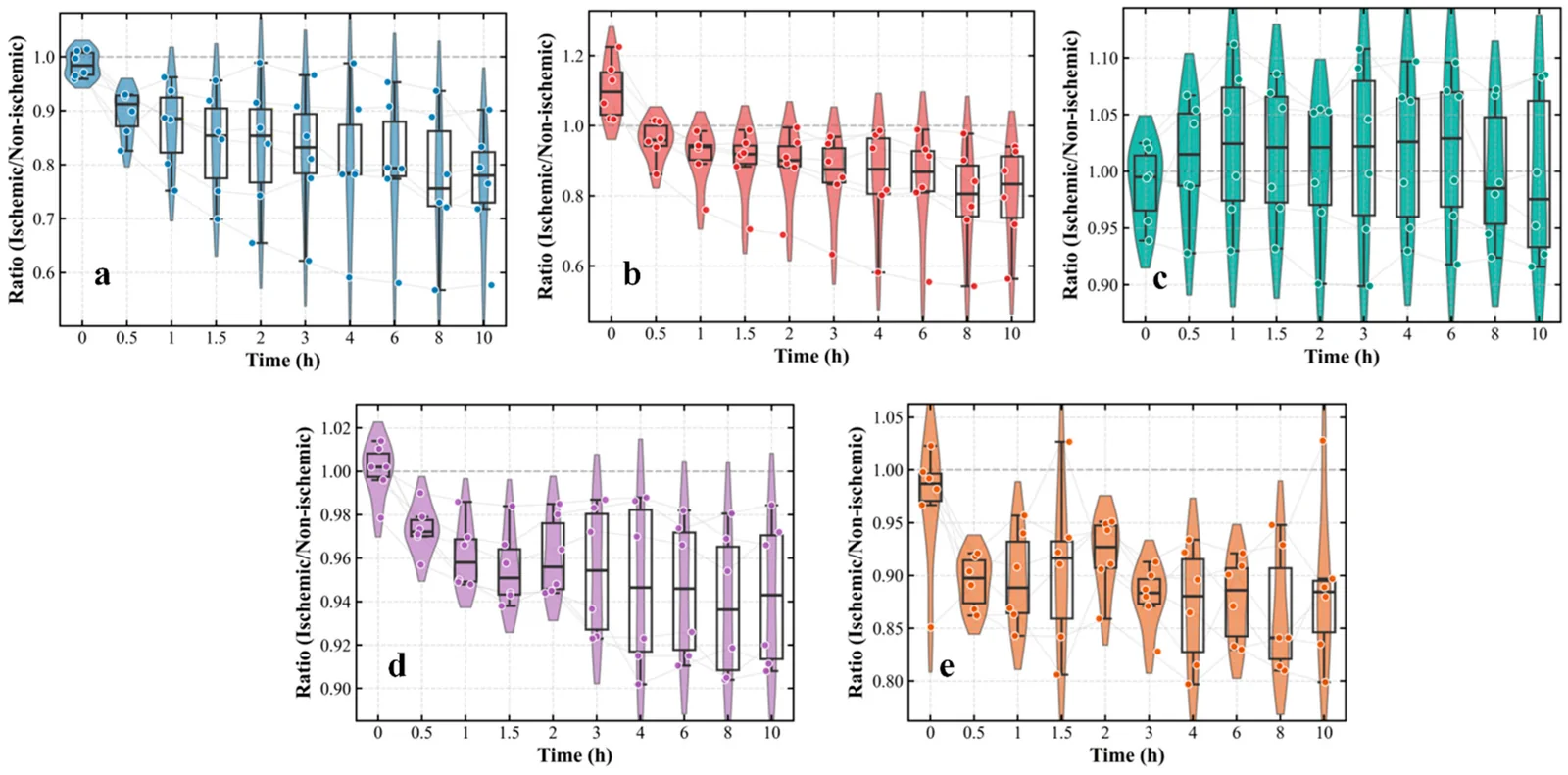
Temporal evolution of quantitative parameter ratios between the ischemic and non-ischemic hemispheres: (**a**) VPD ratio, (**b**) VL ratio, (**c**) VAD ratio, (**d**) AC ratio, (**e**) BPD ratio. Violin shapes indicate the descriptive distribution of the six animal-level ratios, internal boxes indicate the interquartile range, central lines indicate the median, and colored points represent individual mice. Gray connecting lines show paired trajectories from the same mouse.

To further reduce the influence of inter-individual baseline vascular differences, the ratio of each parameter between the ischemic and non-ischemic hemispheres was calculated (Figure 9).

The VPD and VL ratios after stroke induction decreased and remained below their baseline values, suggesting sustained reductions in OCTA-detectable perfusion coverage and apparent vessel length in the ischemic hemisphere relative to the non-ischemic hemisphere. BPD and AC ratios also showed gradual decreases, indicating altered apparent network organization in the segmented OCTA vascular maps. In contrast, the VAD ratio remained relatively stable with only slight fluctuations. Together, these ratio-based results show that ischemia-related hemispheric differences are mainly reflected in perfusion- and topology-related OCTA biomarkers rather than in average vessel diameter.

**Figure 10:**
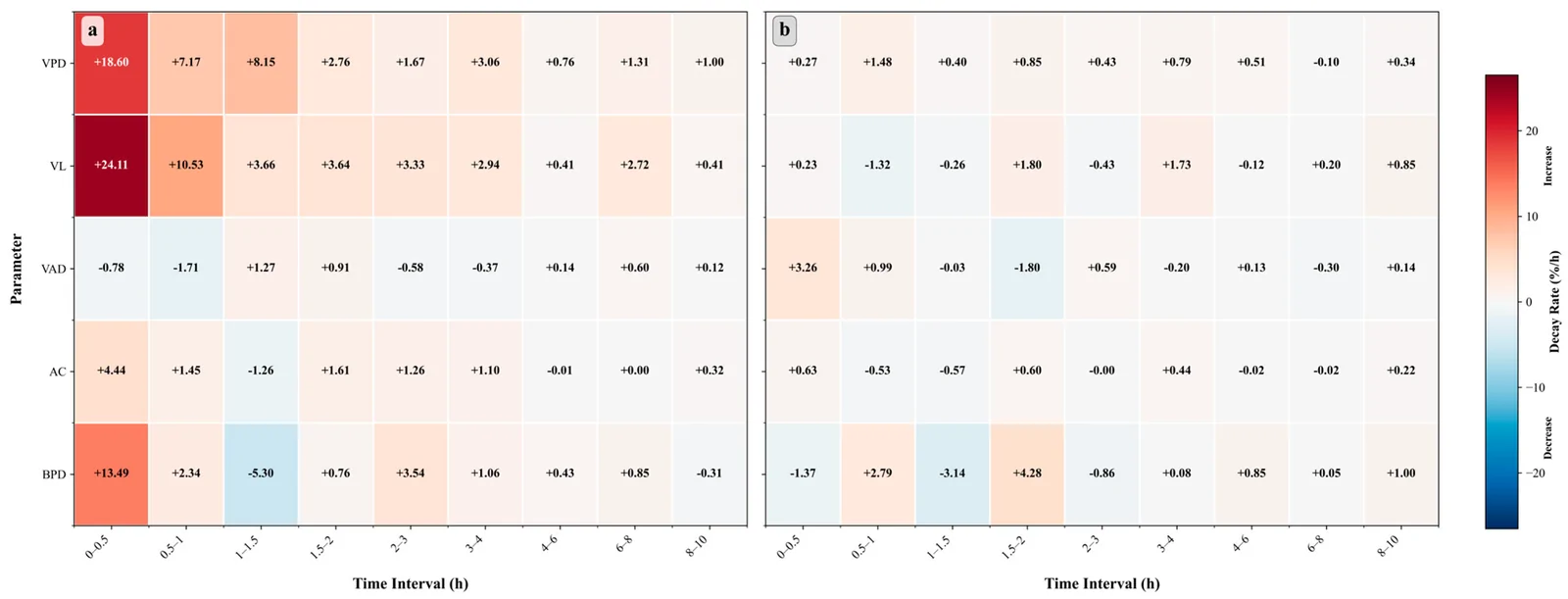
(**a**) Heatmaps illustrating temporal decline rates of quantitative metrics in the ischemic hemisphere. (**b**) Heatmaps illustrating temporal decline rates of quantitative metrics in the non-ischemic hemisphere.

Relative change rates between adjacent time points were calculated to descriptively characterize stage-specific microvascular change velocities (Figure 10).

In the ischemic hemisphere, VPD and VL exhibited relatively rapid declines during the early phase after induction, followed by gradual slowing and transition into a relatively stable phase. BPD and AC showed sustained but gradual downward trends.

VAD fluctuated minimally across most time windows, without evidence of a persistent unidirectional trend.

The non-ischemic hemisphere showed mild fluctuations during certain intervals but did not exhibit a systematic decay pattern.

These findings indicate that OCTA-derived perfusion-related metrics, particularly VPD and VL, decrease earlier and more consistently than VAD within the current observation window. However, because OCTA signal intensity depends not only on vascular morphology but also on flow velocity, decorrelation signal strength, imaging quality, and motion correction, the observed reduction in VPD should be interpreted as a decrease in the detectable perfused vascular signal rather than direct evidence of anatomical vessel loss.

**Figure 11:**
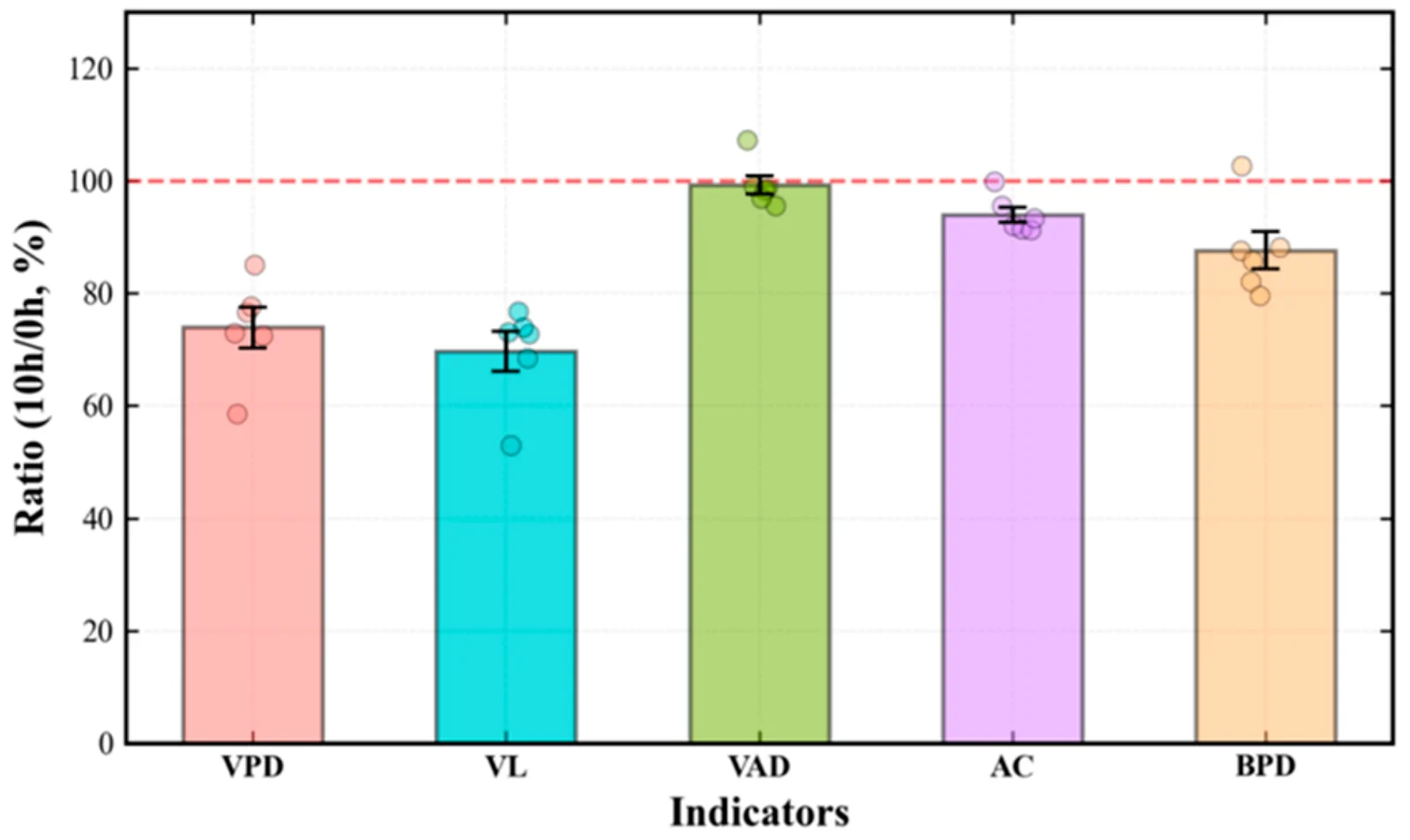
Comparison of vascular parameters in the ischemic hemisphere at baseline and 10 h after ischemia induction. Bars indicate the mean and error bars indicate the standard deviation across six mice. Each dot represents an individual mouse.

At 10 h, VPD and VL remained lower than at baseline (Figure 11). VAD remained stable; the 10 h AC and BPD comparisons were not significant after Holm correction despite significant overall time effects.

The endpoint comparison therefore supports reduced OCTA-detectable coverage and length, but not a uniform reduction across all structural measurements.

Across analyses, the most consistent changes were reduced OCTA-detectable perfusion coverage and apparent vessel length. Diameter changed little, whereas branch- and curvature-based measures were more variable and more dependent on skeleton geometry. The results are limited to this six-animal cohort, imaging window, segmentation pipeline, and OAC-defined ROI.

#### 4.3.4. Robustness of BPD and AC to Segmentation and Skeleton Artifacts

Because BPD and AC depend on local skeleton geometry, sensitivity was tested under component filtering, boundary noise, erosion/dilation, added branches, and centerline deletion. Detailed test data are presented in Table S5 of the Supplementary Materials.

Changing the component-removal threshold from 0 to 200 pixels altered BPD by at most 0.82%/0.39% and AC by 0.29%/0.27% for reference/predicted masks, supporting stability around the 50-pixel setting.

Boundary flips had a larger effect: at 0.5%, BPD changed by 22.10%/20.90% and ICC fell to 0.430/0.460, while AC changed by 4.18%/4.52%. Erosion and dilation also altered both measurements.

Added short branches changed BPD in a dose-dependent manner (about 1.2% at 1% additions to 5.6–6.0% at 5%), whereas AC remained below 1.5%. Centerline deletion affected both measures. BPD was therefore most sensitive to boundary noise and spurious junctions; AC was more affected by morphological and centerline deformation.

#### 4.3.5. Skeletonization Validation and Algorithm-Dependence Analysis

Skeletonization performance was evaluated using synthetic vascular phantoms with known centerlines and paired masks from real OCTA images. The complete results are provided in Supplementary Tables S6 and S7. Zhang–Suen and Lee thinning showed broadly comparable centerline and branch-point recovery, whereas unpruned medial-axis extraction was more sensitive to boundary perturbations and produced less consistent endpoint and branch representations. On real masks, BPD and AC were also relatively consistent between Zhang–Suen and Lee thinning but differed more substantially when medial-axis extraction was used. These findings support Zhang–Suen as a technically appropriate prespecified procedure for the present pipeline, while confirming that BPD and AC remain partly dependent on the skeletonization algorithm and should therefore be interpreted as method-dependent exploratory measurements.

#### 4.3.6. Baseline Repeatability of OCTA-Derived Vascular Measurements

Baseline repeatability was assessed using two independent pre-ischemia acquisitions from each mouse, with the complete results reported in Table S8 of the Supplementary Materials. VPD, VL, and VAD showed relatively high repeatability, with ICCs of 0.90–0.95 and within-subject CVs of 2.4–4.3%, supporting their use in baseline-normalized longitudinal analysis. In contrast, BPD and AC showed greater within-subject variability, consistent with their stronger dependence on local skeleton geometry. Their longitudinal changes were therefore interpreted more cautiously. Because repeatability was evaluated in only six paired acquisitions, these results characterize the stability of the present acquisition and analysis pipeline rather than providing definitive validation of biomarker reproducibility.

## 5. Discussion

### 5.1. Segmentation Performance and Structural Consistency

The proposed framework improved region overlap, average boundary agreement, centerline coverage, and branch-point localization on the fixed internal test subset. It did not lead in every metric: U-Net had higher recall, Swin-Unet had slightly lower HD95, and the connected-component error was comparable. The supported claim is, therefore, improved balance across complementary metrics rather than universal superiority.

Cluster-aware tests supported improvements in Dice, clDice, and branch-point F1. Controlled degradation and sensitivity analyses indicated smaller structural-performance losses and stable training across the tested settings, while ablation identified SBD as the strongest individual component and showed non-additive interactions among modules.

The model required 48.99 million parameters and 44.0 ms per image on an RTX 4060. The Dice score of 0.887 obtained in the present study was higher than the dataset- and layer-specific values of approximately 0.626–0.814 reported for OCTAve and was close to the range of approximately 0.892–0.919 reported for LRNet across the ROSSA and OCTA-500 datasets. These comparisons place the present results within the performance range of contemporary OCTA segmentation methods, although differences in imaging anatomy, acquisition field, annotation protocol, and dataset construction should be considered when comparing studies.

### 5.2. Interpretation of Longitudinal Microvascular Alterations After Ischemic Stroke

Longitudinal masks were generated out of sample, image measurements were aggregated within mouse and time point, and the mouse was the independent unit. VPD and VL showed consistent corrected reductions, VAD remained stable, and AC/BPD showed overall effects with fewer significant individual time points.

OCTA visibility depends on flow velocity, signal strength, thresholding, and motion as well as morphology. Reduced VPD and VL therefore indicate lower OCTA-detectable perfusion coverage and apparent vascular extent, not proven anatomical vessel loss or a causal sequence of degeneration.

### 5.3. Relationship Between Segmentation and Vascular Quantification

The benchmark tested segmentation performance, whereas the longitudinal component used the proposed model as a measurement tool; it did not compare vascular curves from competing models. The two analyses are distinct but sequential because all biomarkers derive from predicted masks.

Targeted perturbation, skeleton-algorithm, and repeat-acquisition analyses showed that BPD and AC were more sensitive than area- and diameter-based measures. Zhang–Suen and Lee were broadly consistent, while medial-axis extraction differed more substantially.

These analyses support the prespecified pipeline within the current dataset but do not constitute complete uncertainty propagation, histological validation, or proof of biomarker reproducibility across devices and populations.

### 5.4. Considerations for Future Clinical Translation

Although the proposed framework was developed in a photothrombotic mouse stroke model, its emphasis on structural consistency may be relevant to future clinical OCTA analysis. Reliable vessel segmentation is essential for extracting quantitative vascular measurements, including perfusion density, vessel length, diameter, branch density, and tortuosity.

At present, however, the framework should be considered a preclinical methodological prototype rather than a clinically deployable system. Human cerebral OCTA presents greater anatomical variability, more complex vascular patterns, stronger motion artifacts, heterogeneous acquisition protocols, and device-dependent signal differences, all of which may affect segmentation performance and biomarker stability. Clinical translation will therefore require validation in larger independent, multicenter, and cross-device datasets, together with further investigation of domain adaptation, device harmonization, and the clinical interpretability of OCTA-derived vascular measurements.

### 5.5. Limitations and Future Perspectives

This study has several limitations. The longitudinal cohort included six male mice, and the internal segmentation test set contained source images from one held-out mouse. Exact animal age was unavailable, and no a priori power calculation was performed. The OAC-defined ROI was not histologically confirmed; expert annotation scores were descriptive rather than based on repeat annotation; the 20-image ROI analysis assessed technical agreement; and BPD/AC depended on skeletonization. Future work should include repeat annotations and scans, histological or multimodal validation, cross-model biomarker comparisons, broader uncertainty analysis, and larger independent datasets.

## 6. Conclusions

This study developed a structure-aware cerebral OCTA segmentation framework integrating SBD, GBAM, MDSS, and MCAM within Swin-LiteMedSAM. The method improved overlap, average boundary, centerline, and branch-point performance under the present internal evaluation and showed smaller losses under controlled degradation.

In a separate six-animal longitudinal application using out-of-sample masks, VPD and VL decreased significantly, VAD remained stable, and AC/BPD showed overall temporal effects with limited corrected time-point differences. These findings represent exploratory, segmentation-derived OCTA measurements rather than histological endpoints.

The framework therefore provides a technically effective approach to cerebral OCTA vessel segmentation and a basis for cautious exploratory quantification after experimental ischemic stroke.

## Figures and Tables

**Table 1:** Comparison between representative vessel-segmentation strategies and the proposed framework. Abbreviation: MedSAM, Segment Anything Model for medical images.

Method/Strategy	Representative Examples	Boundary Modeling	Structure/Centerline Awareness	Multi-Scale Learning	Relevance to Ischemic OCTA Quantification
**Traditional vessel segmentation**	Otsu, Frangi	Not learned	Not explicit	Partial	Sensitive to contrast reduction and signal attenuation
**General deep segmentation models**	U-Net, UNet++, TransUNet, Swin-Unet	Implicit/partial	Not explicit	Yes	Strong region-based baselines, but not specifically designed for ischemic OCTA structural degradation
**Boundary-aware methods**	Boundary loss, contour supervision	Explicit	Partial	Partial	Improves edge localization, but may require additional structural guidance under weak OCTA boundaries
**Topology/skeleton-oriented methods**	Topology-preserving loss, skeleton loss, clDice	Partial	Explicit	Partial	Improves tubular or centerline consistency, but may not fully address boundary uncertainty and low-perfusion signals
**Medical foundation segmentation models**	MedSAM, LiteMedSAM, Swin-LiteMedSAM	Implicit/partial	Not task-specific	Yes	Provides strong representations, but requires OCTA-specific structural adaptation
**Proposed framework**	Swin-LiteMedSAM + SBD + GBAM + MDSS + MCAM	Explicit, task-oriented	Structure-sensitive, not a formal topology guarantee	Yes	Designed for ischemic OCTA segmentation and segmentation-derived vascular quantification

Note: “Explicit” indicates that the corresponding strategy directly introduces this type of constraint. “Partial” indicates indirect or limited consideration. “Structure-sensitive” does not imply a formal guarantee of topology preservation or validated biomarker reproducibility.

**Table 2:** Interobserver consistency of OAC-defined low-attenuation ROI delineation in the prespecified 20-image technical-validation subset. Abbreviations: Dice, Dice similarity coefficient; ICC, intraclass correlation coefficient.

Metric	Value (Mean ± SD)
**Dice**	0.91 ± 0.03
**IoU**	0.84 ± 0.04
**ICC**	0.92 (95% CI: 0.88–0.95)

Note: Dice and IoU values are reported as mean ± standard deviation across the 20 original, non-augmented images. ICC is reported with its 95% confidence interval. The 20 images constituted a technical-validation subset and were not treated as independent biological replicates.

**Table 3:** Quantitative comparison of conventional segmentation metrics using 75 test image instances nested within 25 source OCTA images.

Model	Dice ↑	IoU ↑	Acc ↑	Rec ↑	HD_95_ ↓	ASD ↓
**Otsu**	0.678 ± 0.016	0.515 ± 0.020	0.946 ± 0.010	0.562 ± 0.028	69.607 ± 15.375	1.931 ± 0.071
**U-Net**	0.801 ± 0.005	0.718 ± 0.005	0.857 ± 0.008	**0.938 ± 0.008**	36.138 ± 12.822	0.723 ± 0.054
**UNeXt**	0.848 ± 0.006	0.735 ± 0.010	0.967 ± 0.002	0.879 ± 0.007	26.255 ± 10.514	0.841 ± 0.039
**HiFormer**	0.831 ± 0.005	0.711 ± 0.008	0.944 ± 0.004	0.844 ± 0.014	27.410 ± 6.298	0.975 ± 0.038
**Swin-Unet**	0.855 ± 0.006	0.747 ± 0.008	0.969 ± 0.002	0.895 ± 0.010	**22.310 ± 4.981**	0.745 ± 0.022
**TransUNet**	0.847 ± 0.007	0.735 ± 0.010	0.966 ± 0.002	0.916 ± 0.004	24.410 ± 9.130	0.805 ± 0.011
**Ours**	**0.887 ± 0.002**	**0.797 ± 0.004**	**0.983 ± 0.001**	0.924 ± 0.005	22.540 ± 9.420	**0.564 ± 0.030**

Note: The best results for each metric are highlighted in bold, while the second-best results are underlined. ↑ indicates that a higher value is better, whereas ↓ indicates that a lower value is better.

**Table 4:** Quantitative comparison of topology- and centerline-specific metrics using the same 75 test image instances nested within 25 source OCTA images. Abbreviations: CC, connected component; clDice, centerline Dice coefficient.

Model	clDice ↑	Centerline Recall ↑	Branch-point F1 ↑	CC Relative Error ↓	Centerline Precision ↑
**Otsu**	0.748 ± 0.078	0.638 ± 0.127	0.361 ± 0.086	1.101 ± 0.679	0.904 ± 0.039
**U-Net**	0.941 ± 0.004	0.968 ± 0.009	0.614 ± 0.026	0.610 ± 0.043	0.914 ± 0.002
**UNeXt**	0.892 ± 0.005	0.900 ± 0.008	0.522 ± 0.026	0.616 ± 0.039	0.890 ± 0.003
**HiFormer**	0.895 ± 0.005	0.886 ± 0.013	0.557 ± 0.019	0.699 ± 0.044	0.904 ± 0.007
**Swin-Unet**	0.925 ± 0.007	0.959 ± 0.014	0.699 ± 0.016	**0.196 ± 0.023**	0.896 ± 0.019
**TransUnet**	0.912 ± 0.009	0.949 ± 0.007	0.540 ± 0.038	0.263 ± 0.019	0.878 ± 0.011
**Ours**	**0.953 ± 0.004**	**0.981 ± 0.002**	**0.739 ± 0.011**	0.201 ± 0.020	**0.922 ± 0.003**

Note: The best results for each metric are highlighted in bold, while the second-best results are underlined. Upward arrows indicate that higher values represent better performance, whereas downward arrows indicate that lower values represent better performance.

**Table 5:** Cluster-aware paired statistical comparison between the proposed method and the strongest competing method using 75 test image instances nested within 25 original OCTA source images. Abbreviation: CI, confidence interval.

Metric	Friedman χ^2^(6)	Overall *p*	Kendall’s W	Best-Performing Comparator	Paired Improvement (95% CI)	Raw *p*	Holm-Adjusted *p*	Rank-Biserial r
**Dice**	95.846	<0.001	0.639	Swin-Unet	0.032 [0.023, 0.040]	2.6 × 10^−5^	3.60 × 10^−5^	0.871
**clDice**	117.034	<0.001	0.780	U-Net	0.012 [0.006, 0.018]	0.00115	0.00140	0.711
**Branch-point F1**	124.149	<0.001	0.828	Swin-Unet	0.040 [0.025, 0.054]	2.17 × 10^−4^	2.40 × 10^−4^	0.791
**CC Relative Error**	117.257	<0.001	0.782	Swin-Unet	−0.005 [−0.013, 0.002]	0.491	0.491	−0.163

Note: The 75 test instances were averaged within 25 source-image identifiers before inference. Friedman tests compared seven methods; two-sided Wilcoxon signed-rank tests compared the proposed method with each baseline, with Holm correction within each metric. Positive paired improvements favor the proposed method. Confidence intervals used 10,000 source-image bootstrap resamples; r is matched-pairs rank-biserial correlation.

**Table 6:** Prompt robustness under bounding-box perturbation.

Prompt Setting	Dice	ASD
**Full bbox**	0.887 ± 0.002	0.564 ± 0.030
**Bbox − 10%**	0.886 ± 0.003	0.615 ± 0.041
**Bbox + 10%**	0.887 ± 0.003	0.567 ± 0.032
**Jittered bbox**	0.880 ± 0.003	0.579 ± 0.030

**Table 7:** Results of ablation experiments.

Model	Component				Index					
	1 ^a^	2 ^b^	3 ^c^	4 ^d^	Dice ↑	IoU ↑	Acc ↑	REC ↑	HD_95_ ↓	ASD ↓
**Baseline**	-	-	-	-	0.8478	0.7358	0.9664	0.9042	33.89	0.712
**Model1**	√	-	-	-	0.8800	0.7861	0.9810	0.9216	25.64	0.588
**Model2**	-	√	-	-	0.8547	0.7322	0.9749	0.9166	31.74	0.694
**Model3**	-	-	√	-	0.8494	0.7155	0.9734	0.9211	29.36	0.672
**Model4**	-	-	-	√	0.8434	0.7100	0.9718	0.9206	33.90	0.900
**Model5**	√	√	-	-	0.8595	0.7318	0.9761	0.9139	32.21	0.762
**Model6**	-	√	√	-	0.8602	0.7330	0.9763	0.9170	28.91	0.625
**Model7**	-	-	√	√	0.8484	0.7150	0.9730	0.9252	33.18	0.883
**Model8**	-	√	-	√	0.8433	0.7059	0.9715	0.9331	37.53	0.906
**Model9**	√	-	√	-	0.8490	0.7150	0.9732	0.9274	32.22	0.760
**Model10**	√	-	-	√	0.8563	0.7337	0.9752	0.9186	31.94	0.754
**Model11**	√	√	-	√	0.8517	0.7192	0.9739	0.9232	33.69	0.799
**Model12**	-	√	√	√	0.8500	0.7166	0.9733	0.9291	34.05	0.845
**Ours**	√	√	√	√	0.8869	0.7968	0.9827	0.9207	22.54	0.563

Note: ↑ indicates that a higher value is better, whereas ↓ indicates that a lower value is better. “√” indicates that the corresponding module or component was included. ^a^ 1: Structure-guided boundary distillation (SBD, Module 1). ^b^ 2: Geometry-aware boundary attention mechanism (GBAM, Module 2). ^c^ 3: Multi-scale deep supervision strategy (MDSS, Module 3). ^d^ 4: Endogenous multi-scale collaborative attention module (MCAM, Module 4).

**Table 8:** Longitudinal statistical analyses of vascular biomarkers in the ischemic hemisphere.

Biomarker	Greenhouse–Geisser-Corrected RM-ANOVA	Corrected *p* Value	Generalized η^2^ ($\eta_{G}^{2}$)	Significant Comparisons Versus 0 h
**VPD**	(F(3.069, 15.344) = 16.197)	4.78 × 10^−5^	0.449	0.5, 1, 1.5, 2, 3, 4, 6, 8 and 10 h
**VL**	(F(3.113, 15.564) = 18.401)	2.01 × 10^−5^	0.522	0.5, 1, 1.5, 2, 3, 4, 6, 8 and 10 h
**VAD**	(F(2.812, 14.058) = 0.625)	0.601	0.033	None
**AC**	(F(3.139, 15.694) = 8.381)	0.00135	0.331	6 h
**BPD**	(F(2.995, 14.974) = 5.081)	0.0127	0.240	4 h

Note: Greenhouse–Geisser-corrected degrees of freedom and *p*-values are reported for all omnibus repeated-measures analyses. The mouse was the experimental unit (*n* = 6). Baseline-to-post-ischemia comparisons were prespecified, and Holm–Bonferroni correction was applied separately across the nine comparisons within each biomarker.

## Data Availability

The data underlying the results presented in this paper are not publicly available at this time but may be obtained from the authors upon reasonable request.

## Supplementary Materials

The following supporting information can be downloaded at: https://www.mdpi.com/article/10.3390/tomography12080110/s1, Table S1: Scoring criteria for expert annotation quality evaluation based on a 5-point Likert scale; Table S2: Complete robustness results under controlled OCTA image degradation; Table S3: Sensitivity of learnable-coefficient initialization, fixed GBAM parameters, and dynamic loss-weight schedules; Table S4: Post hoc paired comparisons of longitudinal vascular biomarkers versus baseline; Table S5: Sensitivity of BPD and AC to segmentation, post-processing, and skeleton perturbations; Table S6: Validation of centerline recovery on synthetic vascular phantoms; Table S7: Dependence of BPD and AC measurements on the skeletonization algorithm in real OCTA masks; Table S8: Repeatability of OCTA-derived vascular measurements between two independent baseline acquisitions.

## Abbreviations

The following abbreviations are used in this manuscript:

OCTA	Optical coherence tomography angiography
SBD	Structure-guided boundary distillation
GBAM	Geometry-aware boundary attention mechanism
MDSS	Multi-scale deep supervision strategy
MCAM	Multi-scale collaborative attention module
OAC	Optical attenuation coefficient
ROI	Region of interest
VPD	Vascular perfusion density
VL	Vessel length
VAD	Vessel average diameter
AC	Average curvature
BPD	Branch-point density
HD95	95th-percentile Hausdorff distance
ASD	Average surface distance
Dice	Dice similarity coefficient
IoU	Intersection over union
Acc	Accuracy
Rec	Recall
ICC	Intraclass correlation coefficient
CI	Confidence interval
CV	Coefficient of variation
FP32	32-bit floating-point precision
FPS	Frames per second
MACs	Multiply–accumulate operations
FLOPs	Floating-point operations
GT	Ground truth
RM-ANOVA	Repeated-measures analysis of variance
