# Supplementary material for: Structure-Aware OCTA Segmentation for OCTA-Derived Quantitative Analysis of Cerebral Microvascular Perfusion in Ischemic Stroke
Source: Tomography. 2026 Jul 29;12(8):110. doi: 10.3390/tomography12080110 (PMC13517168; doi:10.3390/tomography12080110)
Supplement: Supplementary file 1 [file tomography-12-00110-s001.zip › tomography-4384747-supplementary.pdf]

Supplementary Materials

S1 Expert Annotation Quality Evaluation

To make the expert review of vessel annotations transparent and reproducible, a five-point Likert rubric was defined for annotation accuracy, boundary adherence, and topological rationality. The same criteria were applied to the original, non-augmented OCTA image-mask pairs included in the annotation-quality audit.

Table S1 defines the operational criteria for scores from 5 (optimal) to 1 (unacceptable) in each evaluation dimension. Higher scores require close agreement with visible perfusion signals, accurate boundary placement, and plausible vascular connectivity; lower scores indicate progressively greater omission, false-positive labeling, contour deviation, fragmentation, or spurious junctions. The rubric documents the descriptive expert audit and does not replace objective annotation-reproducibility analysis.

Table S1. Scoring criteria for expert annotation quality evaluation based on a 5-point Likert scale.

Evaluation Dimension	Annotation Accuracy	Boundary Adherence	Topological Rationality
**5 (Optimal)**	Vessel pixels fully match real perfusion signals, with no obvious false-positive or false-negative labeling.	Segmentation contours closely follow OCTA vascular edges without obvious offset.	Vascular connectivity is complete, with no artificial disconnection or spurious cross-linking.
**4 (Good)**	Minor false-positive or false-negative labels are present, involving only very small local regions and not affecting major vascular structures.	Slight local contour offset is present, mainly around weak boundaries or bifurcations, but overall boundary consistency is preserved.	Minor local discontinuities or isolated spurious links are present, but the main vascular topology is preserved.
**3 (Acceptable)**	Local missing microvessels or scattered false labels are present, but the main vascular structure remains identifiable.	Visible contour offset is present in multiple local regions, especially around bifurcations or hypoperfusion areas.	Several vessel breakages or false connections are present, but they do not severely alter the overall network structure.
**2 (Poor)**	Frequent missing vessels or false-positive labels are present in multiple regions and affect the representation of the vascular network.	Frequent or relatively large boundary deviations affect local vessel delineation or apparent vessel caliber.	Frequent vessel fragmentation, missing connections, or spurious junctions substantially alters the apparent vascular network.
**1 (Unacceptable)**	Extensive false-positive or false-negative labeling prevents reliable representation of the principal vascular structures.	Widespread boundary mismatch makes the annotation unsuitable for reliable vascular delineation.	Widespread fragmentation or false connections prevent meaningful representation of the vascular network structure.

S2 Controlled OCTA Image-Degradation Robustness Analysis

To assess robustness to image-quality changes commonly encountered in ischemic OCTA, controlled contrast reduction, Gaussian noise, Gaussian blur, and motion blur were independently applied at mild and severe levels. The experiment used the same fixed 75-image test subset as the primary comparison. Swin-LiteMedSAM, Swin-Unet, and the proposed method were evaluated without retraining and under identical prompting, thresholding, and post-processing settings.

As shown in Table S2, performance decreased with increasing degradation severity for all three methods. The proposed method nevertheless retained higher Dice, clDice, and branch-point F1 values and lower ASD than the comparison models under the tested conditions. Its relative clDice reduction was generally smaller, although severe motion blur remained the most challenging degradation.

Table S2. Complete robustness results under controlled OCTA image degradation.

Model	Degradation	Severity/parameter	Dice	clDice	ASD	Branch-point F1	clDice decrease
**Swin-LiteMedSAM backbone**	Clean	—	0.8478	0.9080	0.7120	0.6600	—
**Swin-LiteMedSAM backbone**	Contrast reduction	Mild, c=0.80	0.8190	0.8671	0.7974	0.6105	4.5%
**Swin-LiteMedSAM backbone**	Contrast reduction	Severe, c=0.60	0.7698	0.7945	0.9968	0.5214	12.5%
**Swin-LiteMedSAM backbone**	Gaussian noise	Mild, σ=0.02	0.8266	0.8771	0.7690	0.6237	3.4%
**Swin-LiteMedSAM backbone**	Gaussian noise	Severe, σ=0.05	0.7885	0.8217	0.9114	0.5544	9.5%
**Swin-LiteMedSAM backbone**	Gaussian blur	Mild, σ=1 pixel	0.8224	0.8708	0.7903	0.6171	4.1%
**Swin-LiteMedSAM backbone**	Gaussian blur	Severe, σ=2 pixels	0.7740	0.8009	0.9683	0.5346	11.8%
**Swin-LiteMedSAM backbone**	Motion blur	Mild, L=5 pixels	0.8164	0.8644	0.8330	0.6039	4.8%
**Swin-LiteMedSAM backbone**	Motion blur	Severe, L=9 pixels	0.7554	0.7791	1.0538	0.5016	14.2%
**Swin-Unet**	Clean	—	0.8550	0.9250	0.7450	0.6990	—
**Swin-Unet**	Contrast reduction	Mild, c=0.80	0.8294	0.8871	0.8195	0.6536	4.1%
**Swin-Unet**	Contrast reduction	Severe, c=0.60	0.7823	0.8168	1.0058	0.5662	11.7%
**Swin-Unet**	Gaussian noise	Mild, σ=0.02	0.8353	0.8963	0.7972	0.6675	3.1%
**Swin-Unet**	Gaussian noise	Severe, σ=0.05	0.7969	0.8399	0.9387	0.6011	9.2%
**Swin-Unet**	Gaussian blur	Mild, σ=1 pixel	0.8311	0.8899	0.8121	0.6571	3.8%
**Swin-Unet**	Gaussian blur	Severe, σ=2 pixels	0.7866	0.8233	0.9909	0.5732	11.0%

**Swin-Unet**	Motion blur	Mild, L=5 pixels	0.8199	0.8788	0.8493	0.6361	5.0%
**Swin-Unet**	Motion blur	Severe, L=9 pixels	0.7618	0.7946	1.0877	0.5382	14.1%
**Ours**	Clean	—	0.8870	0.9530	0.5640	0.7390	—
**Ours**	Contrast reduction	Mild, c=0.80	0.8710	0.9273	0.5978	0.7094	2.7%
**Ours**	Contrast reduction	Severe, c=0.60	0.8400	0.8844	0.6768	0.6540	7.2%
**Ours**	Gaussian noise	Mild, σ=0.02	0.8719	0.9301	0.5922	0.7131	2.4%
**Ours**	Gaussian noise	Severe, σ=0.05	0.8444	0.8939	0.6542	0.6688	6.2%
**Ours**	Gaussian blur	Mild, σ=1 pixel	0.8710	0.9292	0.5978	0.7109	2.5%
**Ours**	Gaussian blur	Severe, σ=2 pixels	0.8409	0.8882	0.6768	0.6577	6.8%
**Ours**	Motion blur	Mild, L=5 pixels	0.8622	0.9149	0.6260	0.6895	4.0%
**Ours**	Motion blur	Severe, L=9 pixels	0.8107	0.8482	0.7670	0.5912	11.0%

Note: Values are mean results over the same fixed 75-image test subset used for the primary model comparison. Each degradation was applied independently to the corresponding clean images. No model was retrained for this analysis. Identical bounding-box prompts, probability thresholds, and post-processing settings were used across models and conditions. The clDice decrease was calculated relative to the corresponding clean value. Higher values indicate better performance for Dice, clDice, and branch-point F1, whereas lower values indicate better performance for ASD.

S3 Parameter and Training-Schedule Sensitivity Analysis

To determine whether the reported performance depended on a specific initialization or manually selected training schedule, one-factor-at-a-time sensitivity experiments were conducted for the learnable coefficients α and λ, the fixed GBAM parameters β and τ, the boundary-loss schedule, and the auxiliary-supervision schedule. Each configuration was repeated with three independent random seeds, while all non-varied settings were fixed at the manuscript defaults.

As shown in Table S3, all tested configurations completed successfully and the between-seed variations in Dice, clDice, ASD, and branch-point F1 were limited. The default setting provided the most balanced structural performance, while nearby initializations and schedules produced comparable but slightly weaker results. This supports the stability of the selected configuration without implying that it is a global optimum.

Table S3. Sensitivity of learnable-coefficient initialization, fixed GBAM parameters, and dynamic loss-weight schedules.

A. Learnable-coefficient initialization sensitivity

Setting	Dice	clDice	ASD↓	Branch-point F1↑	Failed runs	Final α	Final λ
**α_0=λ_0=0.25**	0.8854 ± 0.0015	0.9497 ± 0.0020	0.579 ± 0.014	0.7270 ± 0.0080	0/3	0.612 ± 0.025	0.572 ± 0.024
**α_0=λ_0=0.50, default**	0.8870 ± 0.0012	0.9530 ± 0.0015	0.564 ± 0.011	0.7390 ± 0.0060	0/3	0.642 ± 0.019	0.603 ± 0.018
**α_0=λ_0=0.75**	0.8861 ± 0.0014	0.9512 ± 0.0018	0.572 ± 0.013	0.7330 ± 0.0070	0/3	0.674 ± 0.027	0.6240.024

B. GBAM fixed-parameter sensitivity

Setting	Dice	clDice	ASD↓	Branch-point F1↑	Failed runs
**β=5, τ=0.50**	0.8872 ± 0.0014	0.9508 ± 0.0018	0.574 ± 0.013	0.7310 ± 0.0070	0/3
**β=10, τ=0.50, default**	0.8870 ± 0.0012	0.9530 ± 0.0015	0.564 ± 0.011	0.7390 ± 0.0060	0/3
**β=20, τ=0.50**	0.8856 ± 0.0016	0.9499 ± 0.0021	0.580 ± 0.015	0.7260 ± 0.0090	0/3
**β=10, τ=0.30**	0.8859 ± 0.0015	0.9505 ± 0.0019	0.576 ± 0.014	0.7290 ± 0.0080	0/3
**β=10, τ=0.70**	0.8853 ± 0.0017	0.9494 ± 0.0022	0.582 ± 0.016	0.7240 ± 0.0090	0/3

C. Boundary-loss schedule sensitivity

Setting	Dice	clDice	ASD↓	Branch-point F1↑	Failed runs
**Fixed w_{bd}=0.20**	0.8845 ± 0.0018	0.9466 ± 0.0024	0.593 ± 0.017	0.7140 ± 0.0100	0/3
**Linear 0.20→0.60, default**	0.8870 ± 0.0012	0.9530 ± 0.0015	0.564 ± 0.011	0.7390 ± 0.0060	0/3
**Fixed w_{bd}=0.60**	0.8854 ± 0.0016	0.9493 ± 0.0020	0.579 ± 0.014	0.7260 ± 0.0080	0/3

D. Auxiliary-supervision schedule sensitivity

Setting	Dice	clDice	ASD↓	Branch-point F1↑	Failed runs
**Fixed γ=(0.10,0.10,0.10)**	0.8849 ± 0.0017	0.9472 ± 0.0023	0.589 ± 0.016	0.7170 ± 0.0100	0/3
**Linear high→low, default**	0.8870 ± 0.0012	0.9530 ± 0.0015	0.564 ± 0.011	0.7390 ± 0.0060	0/3
**Fixed γ=(0.30,0.20,0.30)**	0.8873 ± 0.0015	0.9496 ± 0.0019	0.577 ± 0.014	0.7270 ± 0.0080	0/3

Note: Values are reported as mean ± standard deviation across three independent training seeds. Test metrics were first averaged over the fixed test subset within each seed. Therefore, the standard deviations reported here represent between-seed training variability and should not be directly compared with the image-level standard deviations reported in Tables 8 and 9. The manuscript-default configuration was repeated across panels to facilitate comparison. All sensitivity experiments followed a one-factor-at-a-time design, with non-varied parameters maintained at their default values. Higher values indicate better performance for Dice, clDice, and branch-point F1, whereas lower values indicate better performance for ASD.

S4 Longitudinal Post Hoc Statistical Analysis

To identify the post-ischemic time points that differed from baseline after the overall longitudinal tests, prespecified paired comparisons were performed for each OCTA-derived vascular measurement. The mouse was the independent experimental unit (n=6), and each post-ischemic value was paired with its corresponding 0 h value. The nine comparisons within each biomarker were adjusted as one family using the Holm-Bonferroni procedure; unadjusted 95% confidence intervals and Cohen's dz were also reported.

As shown in Table S4, VPD and VL were lower than baseline at all evaluated post-ischemic time points after multiplicity correction, whereas VAD showed no significant pairwise differences. For AC and BPD, only selected time points remained significant after correction. The pairwise evidence was therefore strongest and most consistent for the reductions in OCTA-detectable perfusion coverage and apparent vessel length.

Table S4. Post hoc paired comparisons of longitudinal vascular biomarkers versus baseline.

Biomarker	Comparison	Mean difference	Unadjusted 95% CI	Adjusted p value	Cohen's dz
**VPD**	0.5 h vs 0 h	-0.093	[-0.150946, -0.035048]	**0.033**	-1.684
	1 h vs 0 h	-0.125	[-0.201672, -0.048924]	**0.033**	-1.722

	1.5 h vs 0h	-0.160	[-0.250919, -0.068605]	**0.032**	-1.839
	2 h vs 0 h	-0.170	[-0.279114, -0.060225]	**0.033**	-1.627
	3 h vs 0 h	-0.183	[-0.296251, -0.069955]	**0.033**	-1.698
	4 h vs 0h	-0.211	[-0.318849, -0.102747]	**0.027**	-2.047
	6 h vs 0 h	-0.223	[-0.332096, -0.113377]	**0.027**	-2.137
	8 h vs 0 h	-0.242	[-0.363254, -0.119973]	**0.027**	-2.084
	10 h vs 0h	-0.261	[-0.352711, -0.169230]	**0.007**	-2.985
	0.5 h vs 0 h	-0.121	[-0.187203, -0.053918]	**0.017**	-1.898
	1 h vs 0 h	-0.169	[-0.241486, -0.096473]	**0.013**	-2.446
	1.5 h vs 0h	-0.183	[-0.274490, -0.091216]	**0.017**	-2.094
	2 h vs 0 h	-0.198	[-0.290579, -0.104793]	**0.017**	-2.233
**VL**	3 h vs 0 h	-0.223	[-0.333507, -0.111776]	**0.017**	-2.107
	4 h vs 0h	-0.247	[-0.363364, -0.130346]	**0.017**	-2.224
	6 h vs 0 h	-0.253	[-0.374396, -0.130820]	**0.017**	-2.177
	8 h vs 0 h	-0.295	[-0.406563, -0.182986]	**0.009**	-2.767
	10 h vs 0h	-0.304	[-0.394083, -0.212949]	**0.003**	-3.517
	0.5 h vs 0 h	0.004	[-0.021463, 0.029311]	1.000	0.162
	1 h vs 0 h	0.012	[-0.017047, 0.041999]	1.000	0.443
	1.5 h vs 0h	0.006	[-0.032184, 0.044262]	1.000	0.166
	2 h vs 0 h	0.001	[-0.037873, 0.040638]	1.000	0.037
**VAD**	3 h vs 0 h	0.008	[-0.048307, 0.063574]	1.000	0.143
	4 h vs 0h	0.010	[-0.027726, 0.048141]	1.000	0.282
	6 h vs 0 h	0.007	[-0.030228, 0.044799]	1.000	0.204
	8 h vs 0 h	-0.005	[-0.046366, 0.037121]	1.000	-0.116
	10 h vs 0h	-0.007	[-0.049812, 0.035932]	1.000	-0.170
	0.5 h vs 0 h	-0.058	[-0.114443, -0.001648]	0.091	-1.080
	1 h vs 0 h	-0.077	[-0.128188, -0.025414]	0.079	-1.568
	1.5 h vs 0h	-0.061	[-0.159539, 0.037685]	0.173	-0.648
	2 h vs 0 h	-0.082	[-0.136281, -0.028089]	0.079	-1.594
**AC**	3 h vs 0 h	-0.114	[-0.188756, -0.038684]	0.079	-1.590
	4 h vs 0h	-0.141	[-0.235447, -0.045998]	0.079	-1.559
	6 h vs 0 h	-0.141	[-0.214490, -0.066825]	**0.040**	-1.999
	8 h vs 0 h	-0.141	[-0.257346, -0.024669]	0.079	-1.272
	10 h vs 0h	-0.158	[-0.249366, -0.065748]	0.056	-1.801
	0.5 h vs 0 h	-0.067	[-0.145380, 0.010512]	0.278	-0.908
	1 h vs 0 h	-0.078	[-0.164677, 0.008984]	0.278	-0.941
	1.5 h vs 0h	-0.052	[-0.177402, 0.074240]	0.340	-0.430
	2 h vs 0 h	-0.061	[-0.129958, 0.008203]	0.278	-0.925
**BPD**	3 h vs 0 h	-0.095	[-0.156423, -0.032781]	0.066	-1.606
	4 h vs 0h	-0.106	[-0.157016, -0.054608]	**0.028**	-2.169
	6 h vs 0 h	-0.114	[-0.179211, -0.047963]	0.054	-1.816
	8 h vs 0 h	-0.128	[-0.206371, -0.050311]	0.058	-1.726
	10 h vs 0h	-0.124	[-0.208143, -0.039209]	0.066	-1.537

Note: Mean paired differences were calculated as the post-ischemic time-point value minus the corresponding baseline value. The mouse was the experimental unit (n=6). For each biomarker, the nine baseline-to-post-ischemia comparisons constituted one prespecified comparison family, and p-values were adjusted within that family using the Holm–Bonferroni procedure. The reported 95% confidence intervals were unadjusted and should not be interpreted as simultaneous family-wise confidence intervals. Cohen's d_z was calculated using the mean and standard deviation of the within-animal paired differences.

S5 Validation of Skeleton-Derived Vascular Measurements

S5.1 Perturbation Sensitivity of BPD and AC

Because branch-point density (BPD) and average curvature (AC) are derived from vessel skeletons, their sensitivity to segmentation, post-processing, and centerline perturbations was examined on 30 original, non-augmented images. Ground-truth masks and out-of-sample predicted masks were analyzed separately under changes in component threshold, controlled boundary flips, one-pixel erosion or dilation, added spurious branches, and random skeleton-point deletion. Effects were summarized using absolute relative change and, where applicable, absolute-agreement ICC.

As shown in Table S5, small component-threshold changes and low-level skeleton perturbations produced limited variation, whereas stronger boundary flips and morphological operations caused larger deviations and lower agreement. Stability also decreased as more spurious branches were added or skeleton points were deleted. These findings support the use of BPD and AC under a fixed pipeline but confirm their greater sensitivity to structural processing choices.

Table S5. Sensitivity of BPD and AC to segmentation, post-processing, and skeleton perturbations.

Perturbation	BPD change, GT/Ours (%)	BPD ICC, GT/Ours	AC change, GT/Ours (%)	AC ICC, GT/Ours
**Component threshold: 0 vs 50 px**	0.28 / 0.17	—	0.19 / 0.12	—
**Component threshold: 25 vs 50 px**	0.19 / 0.09	—	0.12 / 0.07	—
**Component threshold: 100 vs 50 px**	0.44 / 0.20	—	0.16 / 0.15	—
**Component threshold: 200 vs 50 px**	0.82 / 0.39	—	0.29 / 0.27	—
**Boundary flip 0.1%**	4.70 / 4.40	0.930 / 0.920	0.82 / 0.90	0.910 / 0.930
**Boundary flip 0.25%**	11.30 / 10.70	0.720 / 0.750	2.05 / 2.22	0.710 / 0.730
**Boundary flip 0.5%**	22.10 / 20.90	0.430 / 0.460	4.18 / 4.52	0.460 / 0.510
**One-pixel erosion**	6.50 / 9.00	0.860 / 0.770	5.80 / 8.90	0.440 / 0.390
**One-pixel dilation**	11.40 / 3.80	0.710 / 0.890	5.40 / 5.10	0.530 / 0.610
**Add spurious branches: 1%**	1.25 / 1.18	0.986 / 0.989	0.31 / 0.28	0.974 / 0.979
**Add spurious branches: 3%**	3.62 / 3.41	0.948 / 0.955	0.86 / 0.79	0.914 / 0.927
**Add spurious branches: 5%**	5.96 / 5.64	0.884 / 0.896	1.46 / 1.35	0.842 / 0.861
**Delete skeleton points: 0.5%**	0.51 / 0.55	0.998 / 0.999	0.79 / 0.86	0.940 / 0.960
**Delete skeleton points: 1%**	1.03 / 1.08	0.996 / 0.997	1.68 / 1.79	0.840 / 0.890
**Delete skeleton points: 2%**	2.12 / 2.20	0.987 / 0.990	3.38 / 3.60	0.720 / 0.760

Note: Values are mean absolute relative changes from the unperturbed ground-truth (GT) and out-of-sample predicted masks. ICC is the absolute-agreement ICC(A, 1); higher values indicate better agreement. Perturbations were tested at the listed levels on 30 original images.

S5.2 Synthetic-Phantom Validation of Centerline Recovery

To assess centerline recovery against a known reference, synthetic vascular phantoms with predefined centerlines were evaluated under clean, mildly degraded, and severely degraded conditions. Zhang-Suen thinning, Lee thinning, and the medial-axis method were compared using skeleton Dice, severe-condition HD95, branch-point F1, and unmatched endpoints per image.

As shown in Table S6, Lee thinning achieved the highest overall centerline-recovery performance, while Zhang-Suen produced closely comparable results and clearly outperformed the medial-axis method under degradation. All methods deteriorated as image quality worsened. The findings support Zhang-Suen as a practical and reproducible choice for the present pipeline, rather than as a universally optimal algorithm.

Table S6. Validation of centerline recovery on synthetic vascular phantoms.

Algorithm	Skeleton Dice, clean	Skeleton Dice, mild	Skeleton Dice, severe	Severe HD95 (px)	Severe Branch-point F1	Severe unmatched endpoints/image
**Zhang–Suen**	0.952 ± 0.018	0.914 ± 0.031	0.792 ± 0.055	4.86 ± 1.21	0.612 ± 0.092	11.8 ± 3.4
**Lee thinning**	0.957 ± 0.016	0.921 ± 0.028	0.801 ± 0.051	4.61 ± 1.10	0.637 ± 0.087	10.9 ± 3.1
**Medial-axis**	0.949 ± 0.020	0.887 ± 0.039	0.735 ± 0.068	9.42 ± 5.87	0.438 ± 0.104	25.6 ± 6.8

S5.3 Dependence on the Skeletonization Algorithm in Real OCTA Masks

To evaluate algorithm dependence in real OCTA masks, 20 original, non-augmented images were analyzed using ground-truth masks and out-of-sample predicted masks separately. BPD and AC obtained with Lee thinning and the medial-axis method were compared with Zhang-Suen thinning, the prespecified method used in the main analysis. Relative differences and absolute-agreement ICC values were calculated for each comparison.

As shown in Table S7, Lee thinning yielded relatively small BPD and AC differences and high agreement with Zhang-Suen for both ground-truth and predicted masks. The medial-axis method produced larger differences and lower ICC values, particularly for predicted masks. This confirms algorithm dependence and supports the consistent use of one prespecified skeletonization procedure throughout the longitudinal analysis.

Table S7. Dependence of BPD and AC measurements on the skeletonization algorithm in real OCTA masks.

Source	Comparison	BPD difference vs Zhang–Suen (%)	BPD ICC(A,1)	AC difference vs Zhang–Suen (%)	AC ICC(A,1)	n
**Ground Truth**	Lee vs Zhang–Suen	−5.8 ± 4.2	0.912	+4.8 ± 3.6	0.906	20
**Ground Truth**	Medial-axis vs Zhang–Suen	+18.6 ± 14.2	0.721	+32.5 ± 18.7	0.552	20
**Ours**	Lee vs Zhang–Suen	−7.2 ± 5.6	0.874	+6.1 ± 4.5	0.879	20
**Ours**	Medial-axis vs Zhang–Suen	+27.4 ± 20.8	0.584	+45.8 ± 27.4	0.391	20

Note: Relative differences were calculated using Zhang–Suen as the reference. Ground-truth and predicted masks were analyzed separately. ICC denotes the absolute-agreement, single-measure intraclass correlation coefficient [ICC(A,1)]. Positive relative differences indicate larger values than Zhang–Suen.

S6 Baseline Repeatability Analysis

To evaluate whether the baseline reference was sufficiently repeatable for longitudinal comparison, two independent baseline OCTA acquisitions from each of the six mice were processed using the same segmentation and quantification pipeline. Repeatability was characterized using absolute-agreement ICC, within-subject coefficient of variation, mean bias, Bland-Altman 95% limits of agreement, and the repeatability coefficient.

As shown in Table S8, VPD, VL, and VAD demonstrated high ICC values (0.90-0.95) and low within-subject variation (2.4%-4.3%). BPD and AC showed lower agreement and greater variability, consistent with their stronger dependence on skeleton structure. Because only six paired baseline observations were available, the confidence intervals were wide and the topology-sensitive results should be interpreted cautiously.

Table S8. Repeatability of OCTA-derived vascular measurements between two independent baseline acquisitions.

Metric	ICC(A,1)	95% CI	Within-subject CV	Bias	95% LoA	Repeatability coefficient
**VPD**	0.92	0.57–0.99	3.6%	−0.005	−0.075 to 0.065	0.070
**VL**	0.90	0.49–0.98	4.3%	−0.011	−0.096 to 0.074	0.085
**VAD**	0.95	0.71–0.99	2.4%	+0.004	−0.041 to 0.049	0.045
**BPD**	0.79	0.20–0.96	9.2%	−0.017	−0.167 to 0.133	0.150
**AC**	0.83	0.31–0.97	7.5%	+0.009	−0.119 to 0.137	0.128

Note: Analyses were based on six paired baseline acquisitions. ICC denotes the absolute-agreement, single-measure ICC [ICC(A,1)]. CV denotes the within-subject coefficient of variation. LoA denotes the Bland – Altman 95% limits of agreement. Confidence intervals were expected to be wide because the independent experimental unit was the mouse (n=6).
